# FBXO7 Confers Mesenchymal Properties and Chemoresistance in Glioblastoma by Controlling Rbfox2‐Mediated Alternative Splicing

**DOI:** 10.1002/advs.202303561

**Published:** 2023-10-11

**Authors:** Shangbiao Li, Yanwen Chen, Yuxin Xie, Hongchao Zhan, Yu Zeng, Kunlin Zeng, Li Wang, Ziling Zhan, Cuiying Li, Liqian Zhao, Xiaoxia Chen, Yujing Tan, Zhongyong Wang, Junguo Bu, Ye Song, Fan Deng, Aidong Zhou

**Affiliations:** ^1^ Department of Radiation Oncology Zhujiang Hospital Southern Medical University Guangzhou 510280 China; ^2^ Department of Cell Biology School of Basic Medical Science Southern Medical University Guangzhou 510515 China; ^3^ Department of Neurosurgery Nanfang Hospital Southern Medical University Guangzhou 510515 China; ^4^ Department of Neurosurgery The Second Affiliated Hospital of Soochow University Suzhou 215004 China; ^5^ Guangdong Province Key Laboratory of Molecular Tumor Pathology Southern Medical University Guangzhou 510515 China

**Keywords:** alternative splicing, chemoresistance, FBXO7, glioblastoma, mesenchymal transformation

## Abstract

Mesenchymal glioblastoma (GBM) is highly resistant to radio‐and chemotherapy and correlates with worse survival outcomes in GBM patients; however, the underlying mechanism determining the mesenchymal phenotype remains largely unclear. Herein, it is revealed that FBXO7, a substrate‐recognition component of the SCF complex implicated in the pathogenesis of Parkinson's disease, confers mesenchymal properties and chemoresistance in GBM by controlling Rbfox2‐mediated alternative splicing. Specifically, FBXO7 ubiquitinates Rbfox2 Lys249 through K63‐linked ubiquitin chains upon arginine dimethylation at Arg341 and Arg441 by PRMT5, leading to Rbfox2 stabilization. FBXO7 controls Rbfox2‐mediated splicing of mesenchymal genes, including *FoxM1, Mta1*, and *Postn*. FBXO7‐induced exon *Va* inclusion of *FoxM1* promotes FoxM1 phosphorylation by MEK1 and nuclear translocation, thereby upregulates CD44, CD9, and ID1 levels, resulting in GBM stem cell self‐renewal and mesenchymal transformation. Moreover, FBXO7 is stabilized by temozolomide, and FBXO7 depletion sensitizes tumor xenografts in mice to chemotherapy. The findings demonstrate that the FBXO7‐Rbfox2 axis‐mediated splicing contributes to mesenchymal transformation and tumorigenesis, and targeting FBXO7 represents a potential strategy for GBM treatment.

## Graphical abstract

The authors discover that FBXO7 confers mesenchymal properties and chemoresistance in glioblastoma. FBXO7 upregulation in mesenchymal glioblastoma stabilizes Rbfox2 through K63‐linked ubiquitination after its arginine dimethylation by PRMT5, and thus controls Rbfox2‐mediated splicing of mesenchymal genes, resulting in glioblastoma stem cell self‐renewal, mesenchymal transformation, and chemoresistance. Targeting the FBXO7‐Rbfox2 axis represents a potential strategy for glioblastoma treatment.

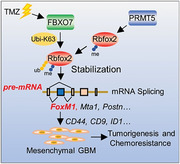

## 1 Introduction

Glioblastoma (GBM) is the most aggressive and lethal form of central nervous system (CNS) tumor and is highly resistant to conventional radio‐and chemotherapy. GBM tumor is composed of heterogeneous tumor cell populations including those with stem cell properties, termed glioblastoma stem cells (GSCs).^[^ ^1^ ^]^ We have previously shown that GSCs play critical roles in GBM initiation, chemo/radio‐resistance, and recurrence.^[^ ^2^ ^]^ Understanding the mechanism regulating GSCs will help to identify novel therapeutic targets and develop more robust and effective therapies for the treatment of GBM.

GBM can be classified into different subtypes based on the molecular signature. There are two biologically distinct GBM subtypes, termed proneural (PN) and mesenchymal (MES), are well recognized and consistent among different classification systems.^[^ ^3^ ^]^ Compared with the PN GBM subtype (PN‐GBM), the MES subtype (MES‐GBM) has a worse prognosis.^[^ ^3^ ^]^ Recent studies support that most GBM subtypes are originated from a common PN‐like precursor glioma, and evolution from PN to MES is considered a major cause of chemo/radio‐resistance, tumor recurrence, and eventual patient death.^[^ ^4^ ^]^ Moreover, radio‐and chemotherapy induce GBM MES transformation, and most recurrent GBM tumors belong to the MES subtype.^[^ ^3^ ^]^ However, little is known about the molecular mechanisms that determine the phenotypic switching during GBM progression and recurrence.

FBXO7, also termed Park15, is a member of the F‐box protein (FBP) family. Like other members of this family, FBXO7 functions as a substrate‐recognition component of the Skp1‐cullin1‐F‐box protein (SCF) ubiquitin E3 ligase complex.^[^ ^5^ ^]^ Besides, FBXO7 also mediates substrate ubiquitination through a SCF‐independent manner.^[^ ^5^ ^]^ FBXO7 is critical for neuronal growth and development. Knockout of FBXO7 in mice is embryonically lethal as neurons die prematurely.^[^ ^6^ ^]^ Mutation of FBXO7, which destabilizes FBXO7 protein,^[^ ^7^ ^]^ is frequent in Parkinson's disease (PD) and is the main cause of early‐onset PD.^[^ ^8^ ^]^ Recent studies support that mutation or deficiency of FBXO7 will lead to mitochondrial dysfunction and blockage of mitophagy, which contribute to the pathogenesis of PD.^[^ ^9^ ^]^ There are only a few reports of FBXO7 in cancer, and the results are often contradictory. FBXO7 has been reported to be highly expressed in lung and colorectal cancer and to promote Cyclin D/Cdk6‐dependent fibroblast transformation.^[^ ^10^ ^]^ On the other hand, FBXO7 is required for genome stability and inhibits the development and progression of colorectal cancer.^[^ ^11^ ^]^

Epidemiological studies have indicated an inverse association between neurodegenerative disease (ND) and CNS tumors.^[^ ^12^ ^]^ This may involve an extreme difference in cell survival: PD is caused by premature cell death, while cancer is caused by uncontrolled cell growth.^[^ ^12a^ ^]^ Although a few genes involved in PD pathogenesis have been shown to regulate the emergence of GBM,^[^ ^13^ ^]^ the mechanism underlying the association between PD and GBM remains largely unknown. In this study, we have identified FBXO7 as a critical regulator of GBM tumorigenesis, MES transformation, and chemoresistance. We found that FBXO7 stabilizes Rbfox2 through K63‐linked ubiquitination and controls Rbfox2‐mediated alternative splicing of mesenchymal genes, which thus promotes GBM MES transformation and chemoresistance. Targeting the FBXO7‐Rbfox2 axis represents a potential strategy against GBM.

## 2 Results

### 2.1 Depletion of FBXO7 Attenuates MES GBM Phenotype and Inhibits Tumorigenesis

**Figure 1:**
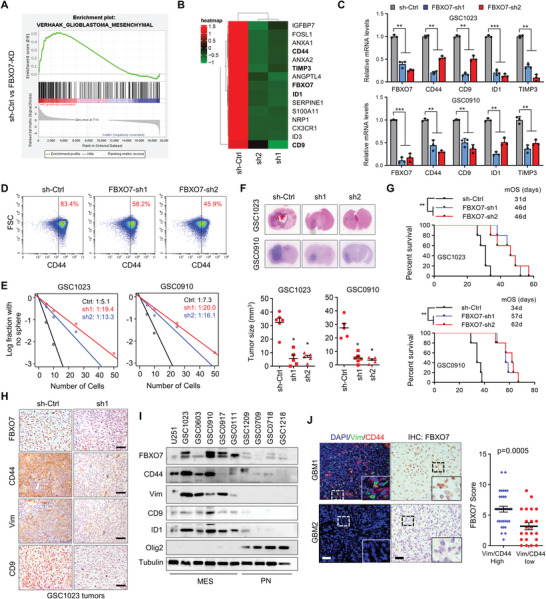
Depletion of FBXO7 represses MES‐GBM properties, GSC self‐renewal, and tumorigenesis. A) Gene Set Enrichment Analysis (GSEA) to discern changes in expression levels of sets of genes after FBXO7 depletion. GSC1023 cells were transfected with two independent shRNAs against *FBXO7*, and high‐throughput transcriptome sequencing was performed. Fold change>1.5 was significant. B) Heatmap shows the change of MES‐related gene expression after FBXO7 depletion in GSC1023 cells. C) The mRNA levels of *FBXO7*, *CD44*, *CD9*, *TIMP3*, and *ID1* were determined by RT‐PCR in GSC1023 and GSC0910 cells stably expressing *FBXO7* shRNAs. Values were normalized to control shRNA (mean ± S.E.M., *n* = 3 independent experiments, two‐tailed Student's *t*‐test). GAPDH was used as an internal control. **P<0.05, ***P<0.01. D) Fluorescence‐activated cell sorting (FACS) detected the expression of CD44 on GSC1023 cells after FBXO7 depletion. E) Limiting dilution assays (LDAs) demonstrated the frequencies of neurosphere formation of GSCs after FBXO7 depletion. The frequencies of neurosphere formation were calculated as 1:χ, where χ indicates the average cell number. The significance of the difference between control shRNA and *FBXO7* shRNAs was determined by χ^2^ test (*n* = 3 independent experiments). F) GSC1023 and GSC0910 cells (5 × 10^5^ cells per mouse) stably expressing control or *FBXO7* shRNAs were intracranially injected into nude mice. Thirty days after injection, the mice were humanely killed, and tumor growth was assessed. The H&E‐stained sections show representative tumor xenografts. Tumor volumes were calculated (mean ± S.D., *n* = 5 mice for each group, One‐way ANOVA test). *P<0.05. G) GSC1023 and GSC0910 cells expressing sh‐Ctrl or *FBXO7* shRNAs were intracranially injected into nude mice as above. The survival of mice was evaluated (*n* = 5 mice per each group, Kaplan–Meier model with two‐sided log‐rank test). *P* values were analyzed by comparing FBXO7 shRNAs versus sh‐Ctrl.**P<0.01. H) Consecutive sections from the xenograft tumors of GSC1023 cells were immunostained using the indicated antibodies. Representative images of immunostaining for each group are shown. Scale bar, 100 µm. I) The expression of FBXO7, CD44, CD9, Vimentin, ID1, and Olig2 in a panel of patient‐derived GSCs was detected by immunoblotting. Tubulin was used as a loading control. J) Consecutive sections of 87 human GBM tissues were double‐stained with anti‐CD44 & Vim antibodies, and immunochemically stained with an anti‐FBXO7 antibody, respectively. Representative images of two tumors are shown (left panel). Insets: high magnification images. Scale bar, 50 µm. The levels of CD44 & Vimentin double‐staining and FBXO7 expression were analyzed and compared (right panel, mean ± S.D., *n* = 87 tissues, paired Student's *t*‐test).

The roles of FBXO7 in neuronal growth and development have been well established.^[^ ^14^ ^]^ Both neurons and glial cells are originated from neuron progenitor cells (NPCs). Although mutation of FBXO7, which has been shown to destabilize FBXO7, is frequent in the neurons of Parkinson's disease,^[^ ^7^, ^14^ ^]^ it's rare in GBM (data not shown). To gain insight into the functional significance of FBXO7 in GBM, we depleted FBXO7 in a GBM patient‐derived GSC1023 glioma stem cell (GSC) line and then profiled gene expression by transcriptome sequencing. Gene set enrichment analysis (GSEA) revealed a signature of gene expression changes related to mesenchymal GBM features (**Figure** **1**A; Table S1, Supporting Information), consistent with a most recent finding that FBXO7 promotes epithelial‐to‐mesenchymal transition (EMT) in breast cancer.^[^ ^15^ ^]^ We next detected the expression of the most substantially changed genes that are closely associated with the MES‐GBM phenotype (Figure 1B), including *CD44*, *CD9*, *ID1*, and *TIMP3*, and confirmed that they were all suppressed by FBXO7 depletion (Figure 1C; Figure S1A,B, Supporting Information). FBXO7 depletion on the expression of the MES‐GBM cell surface antigen CD44 was further validated by fluorescence‐activated cell sorting (FACS) assays (Figure 1D). Accordingly, overexpression of FBXO7 in GSC0709 and GSC1209 cells increased the levels of those MES‐GBM markers, and decreased the level of Olig2, a PN‐GBM marker (Figure S1C, Supporting Information).

We next investigated the role of FBXO7 in regulating GSC self‐renewal. Depletion of FBXO7 decreased the frequency of in vitro neurosphere formation as determined by limiting dilution assays (LDA) (Figure 1E), and inhibited both primary and secondary sphere formation efficiency of GSCs (Figure S1D, Supporting Information). Using an in vivo intracranial injection mouse model, we further explored the role of FBXO7 in GBM tumorigenesis. All mice intracranially implanted with GSC1023 and GSC0910 cells developed tumors with characteristic glioblastoma features (Figure 1F). However, depletion of FBXO7 in GSCs abrogated tumor formation (Figure 1F), and substantially improved the survival of mice harboring GSC1023‐GBM tumors (median survival duration of 31 days for sh‐Ctrl versus 46 days for FBXO7‐sh1 & FBXO7‐sh2) and GSC0910‐GBM tumors (34d for sh‐Ctrl versus 57d & 62d for FBXO7‐sh1 & FBXO7‐sh2, respectively) (Figure 1G). Immunohistochemical (IHC) staining showed that the levels of CD44, CD9, and Vimentin were all downregulated in FBXO7‐depleted tumors (Figure 1H). These results suggest that FBXO7 is required for GSC self‐renewal and GBM tumorigenesis.

To determine the potential value of FBXO7 in predicting MES‐GBM phenotype, we detected the expression of FBXO7 protein in a panel of GSCs derived from GBM patients. The GBM cell line U251, which has been shown to possess mesenchymal properties,^[^ ^2b^ ^]^ was used as a control (Figure 1I). FBXO7 was strongly expressed in GSCs with MES‐like properties, including GSC1023, GSC0603, GSC0910, and GSC0917 cells, which had high levels of the MES‐GBM markers, and low level of the PN‐GBM marker Olig2 (Figure 1I). The MES‐like properties of GSC0910 cells and PN‐like properties of GSC0709 cells were further validated by co‐staining of CD44 & Olig2 (Figure S1E, Supporting Information). However, the *FBXO7* mRNA expression wasn't obviously increased in mesenchymal GSCs (Figure S1F, Supporting Information). Consistently, TCGA‐GBM dataset revealed no significant difference in *FBXO7* mRNA levels between MES‐GBM and PN‐GBM (Figure S1G, Supporting Information), suggesting that upregulation of FBXO7 in MES‐GBM may be regulated by a posttranscriptional mechanism. We further determined the expression of FBXO7 in 87 human GBM tissue specimens (Grade IV) by immunostaining, and found that FBXO7 protein levels were positively correlated with the levels of CD44 & Vimentin (Figure 1J). Together, these results indicate that FBXO7 protein is highly expressed in MES‐GBM, and is required for MES phenotype maintenance and GBM tumorigenesis.

### 2.2 FBXO7 Interacts with Rbfox2 and Ubiquitinates Rbfox2 at K249 Through K63‐Linked Ubiquitin Chains

**Figure 2:**
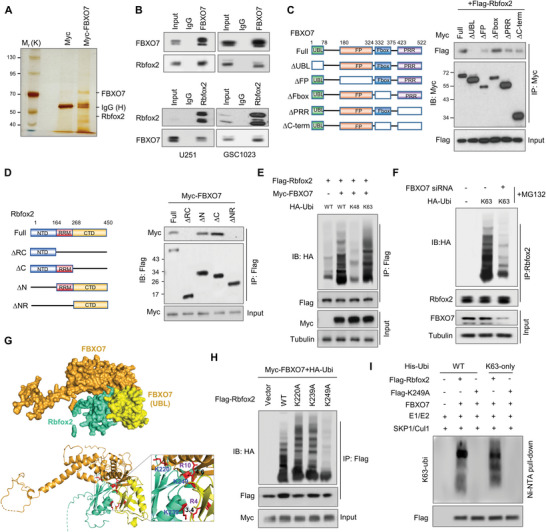
FBXO7 interacts with Rbfox2 and promotes K63‐linked ubiquitination of Rbfox2 at K249. A) U251 cells were transfected with Myc‐FBXO7 and the cell lysate was incubated with an anti‐Myc‐Tag antibody. The immunoprecipitates were resolved on SDS‐PAGE electrophoresis, and specific protein bands, identified by silver staining, were subjected to mass spectrometry analysis. B) U251 and GSC1023 cell lysates were immunoprecipitated using an antibody against FBXO7 or Rbfox2, and then analyzed by immunoblotting. Inputs correspond to 10% cell lysates used for immunoprecipitation. IgG was used as the isotype control. C) Myc‐tagged full‐length FBXO7 or deletion mutants were co‐transfected with Flag‐Rbfox2 into 293T cells. Cell lysates were immunoprecipitated using an anti‐Myc antibody and then were subjected to immunoblotting using an anti‐Flag antibody. D) Flag‐tagged full‐length or deletion constructs of Rbfox2 were co‐transfected with Myc‐FBXO7 into 293T cells. Cell lysates were immunoprecipitated by an anti‐Flag antibody and then analyzed by immunoblotting using an anti‐Myc antibody. E) 293T cells were transfected with Flag‐Rbfox2, Myc‐FBXO7, and HA‐Ubi (WT), HA‐Ubi‐K48 (K48), or HA‐Ubi‐K63 (K63). Cells were treated with MG132 for 6 h before harvest, and cell lysates were immunoprecipitated using an anti‐Flag antibody and then analyzed by immunoblotting. F) GSC1023 cells were transfected with HA‐Ubi‐K63 and FBXO7 siRNA, and cell lysates were immunoprecipitated using an anti‐Rbfox2 antibody and then analyzed by immunoblotting. G) The binding mode of the Rbfox2 and FBXO7 UBL domain was predicted by the HDOCK server. The protein tertiary structures for Rbfox2 and FBXO7 were retrieved from the Alphafold database (Rbfox2: AF‐O43251‐F1; FBXO7: AF‐Q9Y3I1‐F1). The UBL domain of FBXO7 is displayed in yellow and other domains are shown in orange. The RRM domain of Rbfox2 is displayed in cyan (up panel). Rbfox2 and FBXO7 are displayed in ribbon mode from the same angle view as above (lower panel). Interatomic interactions between Rbfox2 bound to the UBL domain of FBXO7 are visualized by PyMOL. Yellow dashed lines indicate possible interactions. Residues highlighted in red indicate involvement in protein‐protein interactions. H) 293T cells were transfected with Myc‐FBXO7, HA‐Ubi, and Flag‐Rbfox2 or each of the Flag‐tagged Rbfox2 mutant (K220A, K239A or K249A). Cell lysates were immunoprecipitated using an anti‐Flag antibody and then analyzed by immunoblotting. I) Purified Flag‐Rbfox2 or Flag‐Rbfox2‐K249A protein was incubated with UBE1, UBE2C, FBXO7, Skp1/Cul1, His‐Ubi/His‐Ubi‐K63, Mg^2+^‐ATP (10 mm) in the ubiquitination reaction buffer. The ubiquitinated proteins were purified by Ni‐NTA beads, and the eluted proteins were analyzed by immunoblotting.

To uncover the underlying mechanism of FBXO7 in regulating MES phenotype and GBM tumorigenesis, we sought to identify the proteins associated with FBXO7. Myc‐tagged FBXO7 was purified from U251 cells and the associated proteins were identified by silver staining and mass spectrometry. Rbfox2 (RBM9), an Rbfox family of RNA splicing factor, was identified as one of the major interacting partners of FBXO7 (**Figure** **2**A; Table S2, Supporting Information). Reciprocal immunoprecipitation (IP) assays confirmed the cellular interaction between FBXO7 and Rbfox2 in both GBM cells and 293T cells (Figure 2B; Figure S2A, Supporting Information). Moreover, FBXO7 and Rbfox2 were co‐localized in the nucleus of GSCs, as determined by IF co‐staining (Figure S2B, Supporting Information). Next, we determined the protein domains mediating the interaction between Rbfox2 and FBXO7 by generating Flag‐tagged and Myc‐tagged truncation mutants, respectively. We found that deletion of the ubiquitin‐like (UBL) domain of FBXO7 (residues 1–78) almost abolished the interaction of FBXO7 with Rbfox2, suggesting that the UBL domain is required for the binding of FBXO7 with Rbfox2 (Figure 2C). Further, deletion of the RNA recognition motif (RRM) (residues 164–268) of Rbfox2 abolished its interaction with FBXO7 (Figure 2D), indicating an indispensable role of the RRM domain in mediating the Rbfox2‐FBXO7 interaction.

FBXO7 is a substrate‐recognizing component of the SCF ubiquitin‐ligase complex, but only a few FBXO7's substrates have been identified. We next investigated the effect of FBXO7 on the ubiquitination of Rbfox2. We found that overexpression of FBXO7 in 293T cells induced Rbfox2 ubiquitination in the presence of the protease inhibitor MG132 (Figure S2C, Supporting Information). Moreover, while transfection of the wild‐type or K63 mutant ubiquitin (Ubi‐K63, K63 wide‐type only) strongly promoted Rbfox2 ubiquitination by FBXO7, other ubiquitin mutants including K6, K11, K27, K29, K33, K48 had no effect on Rbfox2 ubiquitination (Figure 2E; Figure S2D, Supporting Information), indicating that FBXO7 ubiquitinates Rbfox2 through K63‐linked ubiquitin chains. Accordingly, depletion of FBXO7 in GSC1023 cells inhibits K63 ubiquitination of Rbfox2 (Figure 2F). Using a protein‐protein docking model, we found that several lysine residues in the RRM domain of Rbfox2, including K220, K239, and K249, are located at the binding interface between Fbfox2 and FBXO7 and are conserved among different species (Figure 2G; Figure S2E, Supporting Information), suggesting a possible ubiquitination of these residues by FBXO7. Therefore, we mutated these residues respectively (Lys to Ala) and found that K249A almost abolished cellular ubiquitination of Rbfox2 by FBXO7, but not K220A and K239A (Figure 2H). Moreover, in vitro ubiquitination assays using purified proteins showed that FBXO7 ubiquitinated wild‐type Rbfox2 in a K63‐linked manner, but not K249A (Figure 2I). Taken together, these results demonstrate that FBXO7 interacts with Rbfox2 and promotes ubiquitination of Rbfox2 at K249 through K63‐linked ubiquitin chains.

### 2.3 Rbfox2 is Stabilized by FBXO7 and Mediates FBXO7‐Induced MES Phenotype and GBM Tumorigenesis

**Figure 3:**
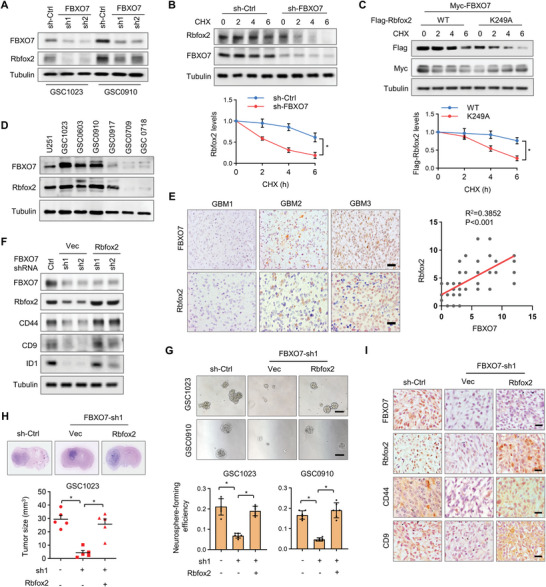
Rbfox2 is stabilized by FBXO7 and mediates FBXO7‐induced MES properties and GBM tumorigenesis. A) GSC1023 and GSC0910 cells stably expressing *FBXO7* shRNAs were analyzed by immunoblotting using the indicated antibodies. α‐tubulin was used as a loading control. B) GSC1023 cells stably expressing control shRNA or *FBXO7* shRNA were treated by CHX for the indicated time intervals, and the cell lysates were subjected to immunoblotting using the indicated antibodies. C) 293T cells were transfected with Myc‐FBXO7 and Flag‐Rbfox2‐WT or Flag‐Rbfox2‐K249A plasmids, and then treated with CHX for the indicated time intervals. Cell lysates were analyzed by immunoblotting. In (B) and (C), the intensities of bands were quantified and the results were expressed as Rbfox2 levels relative to control (mean ± S.D., *n* = 3 independent experiments, paired Student's *t*‐test, right panel). *P<0.05. D) Immunoblotting analysis of Rbfox2 and FBXO7 in U251 cells and different GBM patient‐derived GSCs. E) IHC staining of Rbfox2 and FBXO7 in 87 human GBM specimens. Representative images of three tumors are shown. Scale bar, 100 µm. Staining of Rbfox2 and FBXO7 was scored on a scale of 1–12. The correlation of Rbfox2 and FBXO7 was statistically significant among different specimens (*n* = 87 tissues, *P<*0.001, Pearson correlation test). Note that the scores of some samples overlap. F) GSC1023 cells stably expressing *FBXO7* shRNAs were reconstituted by the expression of Rbfox2, and cell extracts were subjected to immunoblotting analysis using the indicated antibodies. G) Neurosphere formation analysis of GSC1023 and GSC0910 cells stably expressing *FBXO7* shRNA or *FBXO7* shRNA plus Rbfox2. Representative images were shown (up). Scale bar, 500 µm. The neurosphere formation efficiency (spheres/cells plated) was quantified (down, mean ± S.E.M., *n* = 6 independent experiments, two‐tailed Student's *t*‐test). *P<0.05. H) GSC1023 cells stably expressing *FBXO7* shRNA or *FBXO7* shRNA plus Rbfox2 were intracranially injected into nude mice (5 × 10^5^ cells per mouse). Thirty days after injection, the mice were humanely killed and tumor growth was assessed. The H&E‐stained sections show representative tumor xenografts. Tumor volumes were calculated (mean ± S.D., *n* = 5 mice for each group, One‐way ANOVA test). *P<0.05. I) Consecutive sections of tumor xenografts from GSC1023 were immunostained using the indicated antibodies. Representative images for each group were shown. Scale bar, 100 µm.

While K48‐linked ubiquitin chains are major signals for proteasomal degradation, the K63‐Ubi chains are known to modify protein activity, trafficking, or stability.^[^ ^16^ ^]^ We next examined the effect of FBXO7‐mediated K63 ubiquitination on Rbfox2. Unexpectedly, we found that depletion of FBXO7 decreased the level of Rbfox2 in both GSC1023 and GSC0910 cells (**Figure** **3**A), and the effect was reversed by the proteasome inhibitor MG132 (Figure S3A, Supporting Information). While overexpression of FBXO7 in 293T cell promoted Rbfox2 stability (Figure S3B, Supporting Information), FBXO7 depletion in GSCs induced Rbfox2 degradation under treatment by cycloheximide (CHX) (Figure 3B). Further, compared with wild‐type Rbfox2, the K249A mutant was less stable after FBXO7 overexpression (Figure 3C). In a panel of primary cultured GSCs, Rbfox2 was highly expressed in GBM cells with MES‐like properties, and positively correlated with the levels of FBXO7 (Figure 3D). Moreover, immunostaining of 87 human GBM specimens demonstrated that the levels of RBfox2 were positively correlated with FBXO7 levels (Figure 3E). These results support that FBXO7‐mediated K63 ubiquitination of Rbfox2 at K249 induces Rbfox2 stabilization in GBM.

We next investigated the role of Rbfox2 in regulating the mesenchymal properties of GSCs. As with FBXO7 silencing, depletion of Rbfox2 suppressed the expression of CD44, CD9, and ID1 in GSC1023 and GSC0910 cells, as determined by immunoblotting (Figure S3C, Supporting Information). The effect of Rbfox2 depletion on the expression of cell surface CD44 was further confirmed by FACS assays (Figure S3D, Supporting Information). Accordingly, overexpression of Rbfox2 in the PN‐like GSCs increased the levels of the MES‐GBM markers, and decreased the level of Olig2 (Figure S3E, Supporting Information). We next examined the role of Rbfox2 in the neurosphere formation of GSCs. As we expected, we found that depletion of Rbfox2 inhibited in vitro neurosphere formation efficiency of GSCs (Figure S3F, Supporting Information). Moreover, overexpression of Rbfox2 reversed the inhibitory effect of FBXO7 depletion on the expression of CD44, CD9, and ID1 (Figure 3F), and accordingly promoted neurosphere formation of GSCs, which was attenuated by FBXO7 depletion (Figure 3G). In FBXO7‐depleted GSC1023 cells, reconstituted expression of Rbfox2 rescued the effect of FBXO7 depletion on GBM tumorigenesis (Figure 3H). Immunostaining of mouse brain tissues further confirmed that Rbfox2 rescued the expression of downstream MES markers, which were repressed by FBXO7 depletion (Figure 3I). Collectively, these results demonstrate that Rbfox2 is stabilized by FBXO7 and mediates FBXO7‐induced GBM mesenchymal transformation and tumorigenesis.

### 2.4 Rbfox2 is Methylated by PRMT5 at R341 and R441

**Figure 4:**
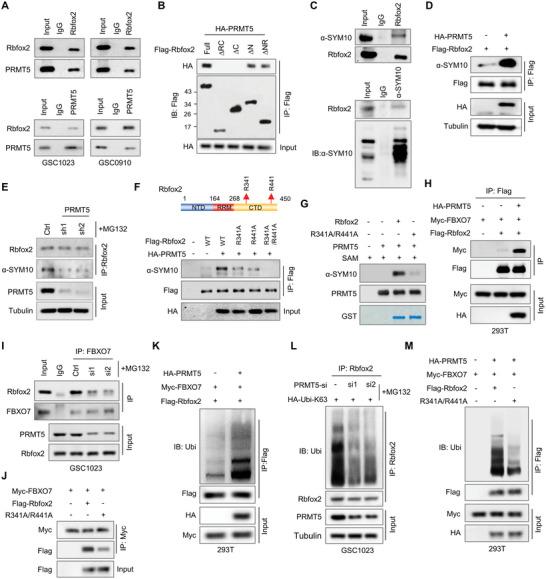
Methylation of Rbfox2 at R341 and R441 by PRMT5 is a prerequisite for its ubiquitination by FBXO7. A) GSC1023 and GSC0910 cell lysates were immunoprecipitated using an antibody against Rbfox2 or PRMT5, and then were subjected to immunoblotting analysis using an anti‐PRMT5 or Rbfox2 antibody, respectively. B) 293T cells were transfected with HA‐PRMT5 and Flag‐tagged wild‐type or different truncation mutants of Rbfox2. Cell lysates were immunoprecipitated with an anti‐Flag antibody, and then analyzed by immunoblotting. C) GSC1023 cell extracts were immunoprecipitated using an anti‐Rbfox2 antibody (up panel) or anti‐dimethyl‐arginine antibody, symmetric (anti‐SYM10), and the resultant precipitates were analyzed by immunoblotting using the anti‐SYM10 or Rbfox2 antibody, respectively. D) 293T cells were transfected with Flag‐Rbfox2 and HA‐PRMT5 plasmids. Cell lysates were immunoprecipitated with an anti‐Flag antibody and then were subjected to immunoblotting using an anti‐SYM10 antibody. E) GSC1023 cells expressing control shRNA or *PRMT5* shRNAs were treated with MG132 for 6 h before cell harvest. Cell lysates were immunoprecipitated using an anti‐Rbfox2 antibody, and then analyzed by immunoblotting using the indicated antibodies. F) 293T cells were transfected with HA‐PRMT5 and Flag‐Rbfox2‐WT, Flag‐Rbfox2‐R341A, Flag‐Rbfox2‐R441A, or Flag‐Rbfox2‐R341A/R441A plasmids. Cell lysates were incubated with an anti‐Flag antibody and then were analyzed by immunoblotting. G) GST‐Rbfox2‐WT or Rbfox2‐R341A/R441A proteins were incubated with purified PRMT5 protein in the reaction buffer, and in vitro methylation of Rbfox2 was detected using an anti‐SYM10 antibody. H) 293T cells were transfected with Myc‐FBXO7, Flag‐Rbfox2, and HA‐PRMT5 plasmids. Cell lysates were immunoprecipitated using an anti‐Flag antibody and then analyzed by immunoblotting using the indicated antibodies. I) GSC1023 cells were transfected with *PRMT5* siRNAs and then were treated with MG132 for 6 h before harvest. The cell lysates were immunoprecipitated using an anti‐FBXO7 antibody, and the resultant precipitates were analyzed by immunoblotting. J) 293T cells were transfected with Myc‐FBXO7 and Flag‐Rbfox2‐WT or Flag‐Rbfox2‐R341A/R441A. Cell lysates were immunoprecipitated using an anti‐Myc antibody and then analyzed by immunoblotting. K) 293T cells were transfected with Myc‐FBXO7, HA‐PRMT5, Flag‐Rbfox2, and HA‐Ubi‐K63. Cell lysates were immunoprecipitated using an anti‐Flag antibody and then analyzed by immunoblotting using the indicated antibodies. L) GSC1023 cells were transfected with *PRMT5* siRNAs and HA‐Ubi‐K63, and then treated with MG132 for 6 h. Cell lysates were immunoprecipitated using an anti‐Rbfox2 antibody, and then analyzed by immunoblotting. M) 293T cells were transfected with Myc‐FBXO7, HA‐PRMT5, HA‐Ubi‐K63, and Flag‐Rbfox2‐wt or Flag‐Rbfox2‐R341A/R441A. Cell lysates were immunoprecipitated using an anti‐Flag antibody and then analyzed by immunoblotting.

Ubiquitination of proteins is usually triggered by other types of modifications, including phosphorylation, acetylation, and methylation. We hypothesized that Rbfox2 ubiquitination might be triggered by another unknown modification. Therefore, we searched for the potential binding partners of Rbfox2 using the Bioplex network of human interactome (https://bioplex.hms.harvard.edu). In addition to FBXO7, PRMT5, a member of the protein arginine methyltransferases (PRMTs) family that catalyzes the symmetric dimethylation of arginine, was shown to potentially interact with Rbfox2 with high probability scores in both HCT116 and 293T cells (Figure S4A, Supporting Information). TCGA datasets showed that *PRMT5* levels are significantly higher in glioma than in normal brain tissues, and high *PRMT5* levels predict poor prognosis in glioma patients (Figure S4B,C, Supporting Information). The cellular interaction between PRMT5 and Rbfox2 was confirmed by reciprocal IP assays in both GSC1023 and GSC0910 cells (**Figure** **4**A). Immunostaining analysis showed that PRMT5 and Rbfox2 were co‐localized in the nucleus (Figure S4D, Supporting Information). Moreover, using a series of deletion constructs of Rbfox2, we found that the C‐terminal domain (CTD) of Rbfox2 was required for its binding with PRMT5 (Figure 4B).

We further determined whether cellular Rbfox2 is methylated by PRMT5. To this end, GSC1023 cell lysates were incubated with an antibody against Rbfox2 or symmetric dimethyl‐arginine (SYM10), and the immunoprecipitates were analyzed by immunoblotting (Figure 4C). As we expected, bands that are specific to methyl‐Rbfox2 were detected in both reciprocal IP/WBs (Figure 4C). Moreover, overexpression of PRMT5 in 293T cells significantly increased the level of methyl‐Rbfox2 (Figure 4D). Accordingly, depletion of PRMT5 in GSC1023 cells decreased the level of methyl‐Rbfox2 (Figure 4E). Proteome‐scale protein modification analysis (PhosphositePlus) revealed that Rbfox2 is methylated at several arginine sites in the CTD domain, including R341, R346, R389, R441, and R446 (Figure S4E, Supporting Information). To determine the residues that are methylated by PRMT5, we mutated each of those residues (Arg to Ala), and found that R341A and R441A decreased Rbfox2 methylation by PRMT5 (Figure S4E, Supporting Information), and R341A/R441A double mutation nearly abolished PRMT5‐mediated methylation of Rbfox2 (Figure 4F). Moreover, in vitro methylation assays using purified proteins showed that PRMT5 methylated wild‐type Rbfox2, but not the R341A/R441A double mutant (Figure 4G). Taken together, these data indicate that PRMT5 interacts with Rbfox2 and methylates Rbfox2 at R341 and R441 in GBM cells.

### 2.5 Methylation of Rbfox2 is a Prerequisite for its Ubiquitination by FBXO7

We next assessed the effect of PRMT5‐mediated methylation of Rbfox2 on its interaction with FBXO7. We found that PRMT5 overexpression in 293T cells promoted the binding between FBXO7 and Rbfox2 (Figure 4H). Accordingly, PRMT5 depletion in GSC1023 cells impaired their interaction (Figure 4I; Figure S4F, Supporting Information). Notably, the R341A/R441A double mutant of Rbfox2 significantly attenuated its interaction with FBXO7 (Figure 4J). Moreover, overexpression of PRMT5 in 293T cells promoted K63‐linked ubiquitination of Rbfox2 (Figure 4K), while PRMT5 depletion in GSC1023 cells inhibited Rbfox2 ubiquitination (Figure 4L). To determine whether the effect of PRMT5 on Rbfox2 ubiquitination depends on the methyltransferase activity, we treated the cells with GSK3326595, a specific inhibitor of PRMT5 methyltransferase activity. We found GSK3326595 almost abolished Rbfox2 ubiquitination that was induced by PRMT5 overexpression (Figure S4G, Supporting Information). Further, the R341A/R441A double mutant greatly decreased K63 ubiquitination of Rbfox2 by FBXO7 (Figure 4M).

Next, we examined the effect of PRMT5 on Rbfox2 expression, and found that PRMT5 depletion decreased the protein level of Rbfox2 in GSCs, but had no effect on *Rbfox2* mRNA (Figure S4H,I, Supporting Information). As with FBXO7 silencing, depletion of PRMT5 promoted the degradation of Rbfox2 in GSCs (Figure S4J, Supporting Information). Because PRMT5‐mediated Rbfox2 methylation is required for Rbfox2 ubiquitination and stabilization by FBXO7, we next explored the potential synergistic effects of FBXO7 silencing plus PRMT5 inhibition on GSC self‐renewal and tumor growth. Compared with GSK3326595 alone, we found that depletion of FBXO7 plus GSK3326595 significantly inhibited the in vitro cell growth and tumorigenicity of GSCs (Figure S4K,L, Supporting Information). Together, these results indicate that PRMT5‐mediated arginine dimethylation of Rbfox2 promotes its binding and ubiquitination by FBXO7.

### 2.6 FBXO7 Controls Rbfox2‐Mediated Alternative Splicing of MES‐Related Genes

**Figure 5:**
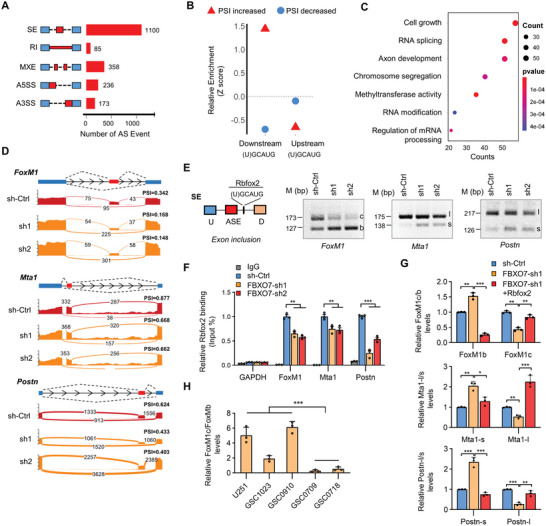
FBXO7 controls Rbfox2‐mediated splicing of mesenchymal genes in GBM cells. A) GSC1023 cells expressing *Rbfox2* shRNAs were analyzed by transcriptome sequencing, and different AS events affected by Rbfox2 depletion were quantified. B) Relative enrichment of the RNA motifs bound by Rbfox2 (UGCAUG) (± 300 bp of the spliced exons). Enrichment scores were computed by comparing Rbfox2‐regulated SEs with control AS events unaffected by Rbfox2. AS events with increased or decreased PSI values upon Rbfox2 depletion were analyzed separately. C) Gene Ontology enrichment scatter plot for Rbfox2‐regulated splicing targets. D) Alternative exons of *FoxM1*, *Mta1*, and *Postn* genes affected by Rbfox2. The numbers of exon junction reads are indicated. E) Semi‐quantitative RT‐PCR (sqRT‐PCR) detected the expression of Rbfox2‐regulated isoforms of *FoxM1*, *Mta1*, and *Postn*. The Rbfox2 motifs (U)GCAUG are located downstream of the spliced exons. *FoxM1c*, *Mta1‐l*, and *Postn‐l* indicated the isoforms of exon‐spliced‐in of the respective genes, whereas *FoxM1b*, *Mta1‐s*, and *Postn‐s* indicated isoforms of exon‐spliced‐out of those genes, respectively. F) RNA immunoprecipitation (RIP) assays determined the binding of Rbfox2 with the indicated pre‐mRNAs after FBXO7 depletion. IgG was used as the isotype control, and *GAPDH* was used as a negative control. Values were expressed as percentages to input. G) GSC1023 cells expressing *FBXO7* shRNA were reconstituted by Rbfox2 expression, and the levels of different splicing variants for each gene were detected by qRT‐PCR respectively. H) The expression of *FoxM1b* and *FoxM1c* in a panel of GSCs was detected by qRT‐PCR. Values were expressed as FoxM1c/FoxM1b levels. *GAPDH* was used as an internal control. From (F) to (H), data were expressed as mean ± S.E.M. of *n* = 3 independent experiments, two‐tailed Student's *t*‐test. **P<0.01, ***P<0.001.

To explore the underlying mechanism of the FBXO7‐Rbfox2 pathway in maintaining GBM mesenchymal properties and tumorigenesis, we next analyzed Rbfox2‐regulated alternative splicing (AS) events. High‐throughput mRNA sequencing of GSC1023 cells after Rbfox2 depletion identified a total of 1952 Rbfox2‐regulated AS events with a significant change of percent‐spliced‐in (PSI) (PSI>0.15) (**Figure** **5**A; Table S3, Supporting Information). In addition to 1100 skipped exons (SEs), Rbfox2 also regulated other types of AS, including alternative 5′ss exons (A5E), alternative 3′ss exons (A3E), retained intron (RI), and mutually exclusive exons (MXE) (Figure 5A; Table S3, Supporting Information). However, Rbfox2 didn't significantly change the overall levels of PSI (Figure S5A, Supporting Information). We next extracted the DNA sequences near the Rbfox2‐regulated SEs (300 bp downstream and upstream, respectively), and the relative abundance of Rbfox2 binding motifs (UGCAUG) located upstream of the SEs was compared with those located downstream (Table S4, Supporting Information). We found that Rbfox2 binding downstream of SEs significantly promoted exon inclusion (PSI increased), whereas Rbfox2 binding upstream of SEs inhibited exon inclusion (PSI decreased) (Figure 5B). This result is consistent with previous findings that the Rbfox2 motifs in the downstream intron generally function to enhance splicing, while upstream motifs tend to inhibit splicing.^[^ ^17^ ^]^

We further evaluated the cellular functions of Rbfox2‐regulated AS events using GO (gene ontology) analysis, and found that the AS genes were enriched in cell growth, RNA splicing, and axon development (Figure 5C; Figure S5B, Supporting Information). These functional enrichment profiles are consistent with previous reports that Rbfox2 regulates RNA processing, splicing, and axon development.^[^ ^18^ ^]^ We next validated the RNA‐seq results by detecting the splicing changes of eight newly identified targets of Rbfox2. We confirmed that Rbfox2 promoted exon inclusion if the binding motif is located downstream of SEs, whereas it inhibited exon inclusion if the motif is located upstream of SEs (Figure 5D,E; Figure S5C,D, Supporting Information). Among the AS genes regulated by Rbfox2, we focused on *FoxM1*, *Mta1* (metastatic tumor antigen 1) and *Postn* (Periostin), which are closely associated with MES properties and tumor progression.^[^ ^2^, ^18^, ^19^ ^]^ The potential Rbfox2 binding motifs in these pre‐mRNAs are all located downstream of the spliced exons, resulting in exon inclusion (Figure 5D,E). We further investigated the effect of FBXO7 on Rbfox2's binding with those target pre‐mRNAs. RNA immunoprecipitation (RIP) assays demonstrated that Rbfox2 specifically bound to the pre‐mRNAs of *FoxM1*, *Mta1*, and *Postn*, and depletion of FBXO7 decreased the levels of these pre‐mRNAs immunoprecipitated by Rbfox2 (Figure 5F). Further, FBXO7 depletion in GSCs inhibited the inclusion of the spliced exons of those genes, and reconstituted expression of Rbfox2 rescued these effects (Figure 5G). Moreover, we found that the relative levels of the transcript variants with exon inclusion were higher in MES‐GSCs than in PN‐GSCs (Figure 5H; Figure S5E, Supporting Information). Together, these results strongly support that the FBXO7‐Rbfox2 axis controls the alternative splicing of mesenchymal genes in GBM.

### 2.7 FBXO7 Maintains MES GBM Phenotype and Promotes Tumorigenesis by Inducing Exon *Va* Inclusion of *FoxM1*

**Figure 6:**
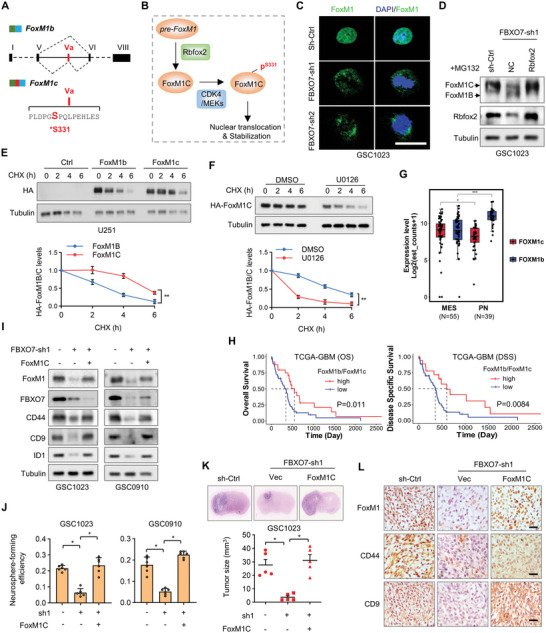
FBXO7 promotes mesenchymal GBM phenotype and tumorigenesis by regulating *FoxM1* splicing. A) Exon‐intron organization of human *FoxM1* isoform b and c. The exon *Va* is included in FoxM1c but excluded in FoxM1b. The amino acid sequence of exon *Va* (15 aa) was indicated, and Ser 331 is a reported phosphorylation site by CDK4/MEK1. B) Scheme shows the proposal for FoxM1 regulation by Rbfox2. FBXO7‐Rbfox2 axis‐mediated *FoxM1* splicing induces *FoxM1c* expression. FoxM1C is phosphorylated by CDK4/MEK1, leading to its nuclear translocation and stabilization. C) IF staining of FoxM1 in GSC1023 expressing *FBXO7* shRNAs. Scale bar, 25 µm. D) GSC1023 cells expressing *FBXO7* shRNA were reconstituted by the expression of Rbfox2. Cells were treated with MG132 for 6 h, and the cell lysates were analyzed by immunoblotting using an anti‐FoxM1 antibody. E) U251 cells expressing FoxM1B or FoxM1C were treated with CHX for the indicated time intervals, and cell lysates were analyzed by immunoblotting. The HA band intensity was quantified. F) U251 cells expressing FoxM1C were treated with MEK1 inhibitor U0126 and then treated with CHX for the indicated time intervals. Cell lysates were analyzed by immunoblotting. The HA‐FoxM1C band intensity was quantified and the results were expressed as levels of DMSO versus U0126. In (E) and (F), data were expressed as mean ± S.D. of *n* = 3 independent experiments, paired Student's *t*‐test. **P<0.01. G) Distribution of FoxM1b and FoxM1c splicing variants in MES‐ and PN‐GBM subtypes using the TCGA‐GBM cohort. *P<0.05, ***P<0.001. H) Kaplan‐Meier overall survival curves show the survival probability of patients (OS and DSS) in TCGA‐GBM cohort, grouped by the ratios of *FoxM1b* and *FoxM1c* expression levels. Patients were grouped based on the best cut‐off of the expression values, determined using the “survminer” package in R software. I) GSC1023 and GSC0910 cells stably expressing *FBXO7* shRNA were reconstituted by FoxM1C expression, and the cell lysates were analyzed by immunoblotting using the indicated antibodies. J) GSC1023 and GSC0910 cells stably expressing *FBXO7* shRNA were reconstituted by FoxM1C expression, and the efficiency of neurosphere formation was assessed (mean ± S.E.M., *n* = 6 independent experiments, two‐tailed Student's *t*‐test). *P<0.05. K) GSC1023 cells expressing *FBXO7* shRNA were reconstituted by FoxM1c expression and then were intracranially injected into nude mice (5 × 10^5^ cells per mouse). The mice were humanely killed and tumor growth was assessed thirty days after injection. The H&E‐stained sections show representative tumor xenografts. Tumor volumes were calculated (mean ± S.D., *n* = 5 mice for each group, One‐way ANOVA test), *P<0.05. L) Consecutive sections of mouse GBM xenografts from GSC1023 cells in (K) were immunostained using the indicated antibodies. Representative images for each group were shown. Scale bar, 100 µm.

Alternative splicing of the transcription factor gene *FoxM1* produces three major isoforms, *FoxM1a*, *b*, and *c*. *FoxM1b* and c are the main isoforms that are expressed in GBM and other cancers.^[^ ^20^ ^]^ Inclusion of exon *Va* (encoding 15 amino acids) produces *FoxM1c*, while exon *Va* exclusion produces *FoxM1b* (**Figure** **6**A). Previous studies have shown that FoxM1 is phosphorylated by CDK4/6 and MEK1 at Ser331, and thus induces FoxM1 nuclear translocation and stabilization.^[^ ^21^ ^]^ Because Ser331 is located in exon *Va*, which is included in the FoxM1C isoform (Figure 6A), we supposed that FoxM1C is more stable and active than FoxM1B (Figure 6B). To this end, we first detected the effect of FBXO7 on FoxM1 expression, and found that FBXO7 depletion in GSCs decreased FoxM1 protein level, but had no effect on *FoxM1* mRNA (Figure S6A,B, Supporting Information). Moreover, depletion of FBXO7 led to increased retention of FoxM1 in the cytoplasm (Figure 6C). In the presence of protease inhibitor MG132, FBXO7 depletion in GSC1023 cells resulted in an increased level of FoxM1B and decreased level of FoxM1C, which were reversed by reconstituted expression of Rbfox2 (Figure 6D). We further detected the cellular localization of those two isoforms and found that FoxM1C was more potent to translocate into nucleus in comparison to FoxM1B (Figure S6C, Supporting Information), and blocking MEK1 activity with U0126 inhibited FoxM1C nuclear localization (Figure S6D, Supporting Information). Moreover, exogenously expressed FoxM1C was more stable than FoxM1B in both U251 and 293T cells (Figure 6E; Figure S6E, Supporting Information), and U0126 treatment induced FoxM1C degradation (Figure 6F). These results suggest that FBXO7‐induced exon *Va* inclusion promotes FoxM1 nuclear translocation and stabilization.

We next investigated the role of FoxM1C in mediating FBXO7‐induced MES transformation and GBM tumorigenesis. TCGA‐GBM dataset showed that the ratios of *FoxM1c and FoxM1b* expression levels (*FoxM1c/FoxM1b)* were significantly higher in MES‐GBM than in PN‐GBM (Figure 6G), and higher *FoxM1c* level in GBM predicted worse patient survival compared with *FoxM1b* (Figure 6H). In patient‐derived GSCs, FoxM1 protein level was higher in MES‐GSCs than in PN‐GSCs (Figure S6F, Supporting Information). Previous studies have shown that CD44, CD9, and ID1 are transcriptionally activated by FoxM1,^[^ ^22^ ^]^ which was confirmed in our study by depleting FoxM1 in GSCs (Figure S6G,H, Supporting Information). Importantly, we found FoxM1C overexpression reversed the inhibitory effect of FBXO7 depletion on the expression of CD44, CD9, and ID1 (Figure 6I). In addition, overexpression of FoxM1C reversed the inhibitory effect of FBXO7 depletion on neurosphere formation of GSCs (Figure 6J). Accordingly, in FBXO7‐depleted GSC1023 cells, reconstituted expression of FoxM1C rescued the effect of FBXO7 depletion on GBM tumorigenesis and on the expression of MES markers in mouse GBM tissues (Figure 6K,L). Together, these results demonstrate that the FBXO7‐Rbfox2 axis‐mediated *FoxM1* splicing maintains mesenchymal GBM properties and promotes tumorigenesis.

### 2.8 Depletion of FBXO7 Sensitizes GBM to Chemotherapy

**Figure 7:**
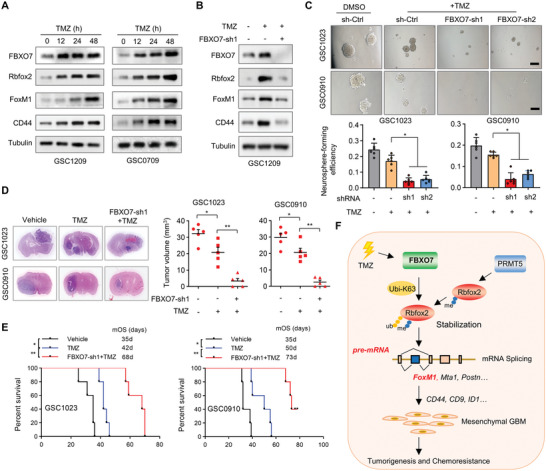
FBXO7 mediates TMZ‐induced mesenchymal properties, and depleting FBXO7 sensitizes GBM to chemotherapy. A) GSC1209 cells were treated with TMZ (100 µm) for the indicated time intervals, and the levels of FBXO7, Rbfox2, FoxM1, and CD44 were detected by immunoblotting. B) GSC1209 cells expressing *FBXO7* shRNA were treated with TMZ for 48 h, and the expression of FBXO7, Rbfox2, FoxM1, and CD44 were detected by immunoblotting. C) GSC1023 and GSC0910 cells expressing *FBXO7* shRNAs or control shRNA were treated with TMZ, and neurosphere formation was assessed. Scale bar, 500 µm. The neurosphere formation efficiency (spheres/cells plated) was quantified (mean ± S.E.M., *n* = 6 independent experiments, two‐tailed Student's *t*‐test). *P<0.05. D) GSC1023 and GSC0910 cells expressing *FBXO7* shRNAs were intracranially injected into nude mice (5 × 10^5^ cells per mouse). One day after cell injection, mice were intraperitoneally injected with TMZ (20 mg kg per d) every other day for 4 weeks. Thirty days after injection, the mice were humanely killed and tumor growth was assessed. The H&E‐stained sections show representative tumor xenografts. Tumor volumes were calculated (mean ± S.D., *n* = 5 mice for each group, One‐way ANOVA test). *P<0.05, **P<0.01. E) GSC1023 and GSC0910 cells were intracranially injected nude mice, and mice were treated as in (D). The survival of mice was evaluated (*n* = 5 mice for each group, Kaplan–Meier model with two‐sided log‐rank test). *P<0.05, **P<0.01. F) Illustration of the FBXO7‐Rbfox2 axis in the regulation of GBM MES transformation, tumorigenesis, and chemoresistance. FBXO7 interacts with and ubiquitinates Rbfox2 through K63‐linked ubiquitin chains after its arginine dimethylation by PRMT5. FBXO7 stabilizes Rbfox2 and controls Rbfox2‐mediated splicing of MES‐related genes, including *FoxM1*, *Mta1*, and *Postn*. FBXO7‐induced exon *Va* inclusion of *FoxM1* induces its nuclear translocation and stabilization, leading to upregulation of CD44, CD9, and ID1, and thus promotes GBM MES transformation and tumorigenesis.

Chemo‐ and radiotherapy can cause a shift in phenotype from PN‐GBM to MES‐GBM, resulting in resistance to those therapies.^[^ ^23^ ^]^ We have previously demonstrated that FoxM1 and CD44 are upregulated by temozolomide (TMZ) in GBM cells.^[^ ^23b^ ^]^ To determine whether the FBXO7‐Rbfox2 axis is involved in this process, we detected their expression in TMZ‐treated GSC1209 and GSC0709 cells. As we expected, besides FoxM1 and CD44, both FBXO7 and Rbfox2 were upregulated by TMZ treatment (**Figure** **7**A). Moreover, FBXO7 depletion reversed the effect of TMZ on the expression of Rbfox2, FoxM1, and CD44 (Figure 7B). Importantly, we found that TMZ upregulated FBXO7 expression by inhibiting its protein degradation but had no obvious effect on *FBXO7* mRNA level (Figure S7A,B, Supporting Information). This result is consistent with our previous finding that FBXO7 protein, but not the mRNA level, was upregulated in MES‐GBM (Figure 1G,H; Figure S1F,G, Supporting Information).

We next assessed the effect of FBXO7 depletion on the sensitivity of MES‐GBM cells to TMZ. We found that TMZ only had a little effect on the viability of GSC1023 and GSC0910 cells, as demonstrated by in vitro neurosphere formation and cell viability assays (Figure 7C; Figure S7C, Supporting Information). Whereas depletion of FBXO7 substantially increased the response of GSCs to TMZ (Figure 7C; Figure S7C, Supporting Information). Using an in vivo intracranial mouse model, we observed that TMZ alone had only a marginal effect on GBM growth (Figure 7D). However, TMZ almost abolished tumor formation of GSCs upon depletion of FBXO7 (Figure 7D). Accordingly, TMZ alone had a modest effect on the survival of GSC1023‐ and GSC0910‐GBM‐bearing mice (Figure 7E). Whereas TMZ plus FBXO7 depletion greatly extended the survival of GSC1023‐GBM‐bearing mice (median survival duration of 35d for sh‐Ctrl versus 68d for FBXO7‐sh1+TMZ), as well as GSC0910‐GBM‐bearing mice (35d for sh‐Ctrl versus 73d for FBXO7‐sh1+TMZ) (Figure 7E). Together, these results indicate that FBXO7 mediates TMZ‐induced MES transformation, and targeting FBXO7 in combination with chemotherapy represents a promising strategy for GBM treatment.

## 3 Discussion

Mesenchymal transformation of GBM is associated with a poor prognosis, and targeting this process represents a promising therapeutic strategy sensitizing tumors to radio‐and chemotherapy. In the current study, we have demonstrated that FBXO7 is highly expressed in MES‐GBM and critical for MES properties and GBM tumorigenesis. We found that FBXO7 stabilizes Rbfox2 through K63‐linked ubiquitination after its arginine dimethylation by PRMT5 and controls Rbfox2‐mediated alternative splicing of MES genes, including *FoxM1*, which thus promotes MES properties and chemoresistance (Figure 7F). Our data consistently supports that the FBXO7‐Rbfox2 axis is critical for GBM mesenchymal transformation, and targeting the axis is a promising strategy for GBM treatment.

Deficiency of FBXO7 induces neuronal death and drives Parkinson's disease, suggesting that FBXO7 is required for neuronal survival and normal differentiation.^[^ ^6^ ^]^ During the preparation of our manuscript, Shen, et al. performed RNAi screening and identified FBXO7 as a critical regulator of mesenchymal phenotype and immune evasion in breast cancer.^[^ ^15^ ^]^ They found that several EMT‐related pathways, including TGF‐β receptor and MAPK signaling, are positively correlated with FBXO7 expression in cancers.^[^ ^15^ ^]^ Although EMT in breast cancer has different characteristics from the PN‐MES transition in GBM, our findings consistently support a requirement of FBXO7 in maintaining MES phenotype. Moreover, of the known ubiquitination targets, FBXO7 tends to ubiquitinate substrates through K63‐linked chains.^[^ ^24^ ^]^ Besides its known functions to modify substrates trafficking and activity, the K63‐linked ubiquitination has been gradually recognized to promote substrate stabilization,^[^ ^16^, ^25^ ^]^ which is further strengthened by our study.

The Rbfox family proteins are highly expressed in brain neurons and regulate the splicing of neuronal transcript.^[^ ^18^ ^]^ Downregulation of the Rbfox family members is frequent in older individuals and contributes to loss of synaptic function in Alzheimer's disease.^[^ ^18^ ^]^ Recent evidences have supported an oncogenic role of Rbfox2 in cancer.^[^ ^26^ ^]^ For example, Rbfox2 mediates TGF‐β‐induced EMT and invasiveness of tumor cells.^[^ ^27^ ^]^ However, the downstream splicing events of Rbfox2 that mediate tumor initiation and EMT remain largely elusive. Our study identified a panel of MES‐related genes as splicing targets of Rbfox2 in GBM, including *FoxM1*, *Mta1*, and *Postn*. Of these target genes, FoxM1 is overexpressed in GSCs, and is required for GBM mesenchymal properties and chemoresistance, as we previously reported.^[^ ^2b^ ^]^ We demonstrated that Rbfox2‐induced splicing of FoxM1 renders its phosphorylation by MEK1, and thus induces its nuclear translocation and stabilization. Therefore, Rbfox2‐mediated splicing of FoxM1 represents a novel posttranscriptional mechanism for FoxM1's upregulation and activation in MES‐GBMs. The clinical significance of *FoxM1* splicing is further strengthened by the facts that the *FoxM1c*/*FoxM1b* ratio is upregulated in MES‐GBM compared with PN‐GBM, and high‐level *FoxM1c* predicts poor survival in GBM (Figure 6g,h).

RNA binding proteins (RBPs) are the most enriched cellular substrates of the protein arginine methyltransferases (PRMTs). Targeting PRMTs leads to inhibition of spliceosome and splicing fidelity, and thereby killing tumors with dysregulated splicing factors.^[^ ^28^ ^]^ PRMTs‐mediated arginine dimethylaltion usually triggers a subsequent modification on substrates, most frequently ubiquitination, and regulates protein activity and stability.^[^ ^29^ ^]^ PRMT5 is a targetable protein in multiple types of cancers.^[^ ^28^, ^30^ ^]^ In GBM, PRMT5 has been reported to mediate GSC stemness and resistance to chemotherapy.^[^ ^31^ ^]^ Besides FBXO7 that has also been identified by IP/MS, we found that PRMT5 is potential binding partner of Rbfox2 with high probability scores using the Bioplex network. We demonstrated that PRMT5 interacts and methylates Rbfox2 at the CTD domain, which triggers subsequent K63 ubiquitination of Rbfox2 by FBXO7, resulting in Rbfox2 stabilization. Recent studies have shown that the CTD domain of Rbfox2 is required for the recruitment of the Large Assembly of Splicing Regulators (LASR) and the splicing activity of Rbfox2.^[^ ^17a^ ^]^ It remains to be identified whether PRMT5‐mediated methylation of Rbfox2 regulates LASR recruitment and splicing activation.

Resistance to chemo‐and radiotherapy usually leads to GBM recurrence, and most recurrent GBM cases show MES phenotype.^[^ ^3^ ^]^ We have demonstrated in this study that FBXO7 protein is upregulated by TMZ and promotes GBM MES transformation. Therefore, FBXO7 may be a key mediator of chemo‐and radiotherapy‐induced PN‐MES transition and acquired resistance to those therapies. Moreover, we found that TMZ induces FBXO7 expression by promoting the protein stability, which may explain the discrepancy between the mRNA and protein abundance of FBXO7 in MES‐ and PN‐GBMs. Recent studies have reported that FBXO7 is a stress‐induced protein, and the amino acids T22, R378, and R498, which are frequently mutated in Parkinson's disease, are associated with FBXO7 stability in a proteasome‐dependent manner.^[^ ^7^, ^32^ ^]^ Further, a chemotherapy‐induced protein, Pink1, has been shown to stabilize FBXO7.^[^ ^33^ ^]^ Thus, it will be interesting to determine the role of those sites in mediating FBXO7 stabilization and upregulation during GBM MES transformation and acquired chemoresistance.

In conclusion, we demonstrate that FBXO7 stabilizes Rbfox2 through K63‐linked ubiquitination, and thus promotes Rbfox2‐mediated alternative splicing of MES genes and GBM MES transformation. Depletion of FBXO7‐Rbfox2 axis inhibits GBM tumorigenesis and sensitizes GBM xenograft to chemotherapy, indicating a potential strategy for the treatment of GBM.

## 4 Experimental Section

Animal experiments were approved by the Ethics Committee for Animal Research of Southern Medical University. Human tissues were obtained with written consent forms and the project was approved by the medical ethics review committee at the Nanfang Hospital of Southern Medical University. The detailed Experimental Section for the cell cultures, GBM tissues, immunoprecipitation, LDA assays, WB, qRT‐PCR, RNA‐seq, histology and immunohistochemistry, and statistical analysis can be found in the Supporting Information.

Significance Statement: Epidemiological studies had indicated an inverse association between neurodegenerative disease and central nervous system tumors, but the underlying mechanism is largely unknown. FBXO7, which was critically implicated in the pathogenesis of Parkinson's disease, confers mesenchymal properties and chemoresistance in glioblastoma. FBXO7 upregulation in MES glioblastoma stabilizes Rbfox2 through K63‐linked ubiquitination after its arginine dimethylation by PRMT5, and thus controls Rbfox2‐mediated splicing of mesenchymal genes, resulting in glioblastoma stem cell self‐renewal, mesenchymal transformation, and chemoresistance. Targeting the FBXO7‐Rbfox2 axis represents a potential strategy for glioblastoma treatment.

## Conflict of Interest

The authors declare no conflict of interest.

## Author Contributions

S.L., Y.C., Y.X., and H.Z. contributed equally to this work. A.Z. conceived the study. S.L., Y.C., and Y.X. designed and performed most of the experiments. H.Z. assisted in the bioinformatic analysis. Y.Z., K.Z., C.L., and L.Z. assisted in some in vitro experiments. L.W., Z.Z., and X.C. assisted in the mouse experiments. Y.T., Z.W., J.B., Y.S., and F.D. provided tissue samples, cell lines, reagents, and/or conceptual advice. A.Z. and S.L. wrote and revised the manuscript. A.Z. supervised the study. All authors discussed the results and commented on the manuscript.

## Supporting information

Supporting Information

Supplemental Table 1

Supplemental Table 2

Supplemental Table 3

Supplemental Table 4

## Data Availability

The data that support the findings of this study are available from the corresponding author upon reasonable request.
