# Supplementary material for: FBXO7 Confers Mesenchymal Properties and Chemoresistance in Glioblastoma by Controlling Rbfox2‐Mediated Alternative Splicing
Source: Adv Sci (Weinh). 2023 Oct 11;10(33):2303561. doi: 10.1002/advs.202303561 (PMC10667838; doi:10.1002/advs.202303561)
Supplement: Supplementary file 1 — Supporting Information [file ADVS-10-2303561-s001.pdf]

ADVANCED SCIENCE
Open Access

Supporting Information

for *Adv. Sci.*, DOI 10.1002/advs.202303561

FBXO7 Confers Mesenchymal Properties and Chemoresistance in Glioblastoma by Controlling Rbfox2-Mediated Alternative Splicing

*Shangbiao Li, Yanwen Chen, Yuxin Xie, Hongchao Zhan, Yu Zeng, Kunlin Zeng, Li Wang, Ziling Zhan, Cuiying Li, Liqian Zhao, Xiaoxia Chen, Yujing Tan, Zhongyong Wang, Junguo Bu, Ye Song, Fan Deng and Aidong Zhou**

Supporting Information for

FBXO7 Confers Mesenchymal Properties and Chemoresistance in Glioblastoma by Controlling Rbfox2-Mediated Alternative Splicing

Shangbiao Li[1,2#], *Yanwen Chen*[2#], *Yuxin Xie*[2#], *Hongchao Zhan*[2#], *Yu Zeng*[2], *Kunlin Zeng*[2], *Li Wang*[2], *Ziling Zhan*[2], *Cuiying Li*[2], *Liqian Zhao*[3], *Xiaoxia Chen*[2], *Yujing Tan*[1], *Zhongyong Wang*[4], *Junguo Bu*[1], *Ye Song*[3], *Fan Deng*[2], *Aidong Zhou*[1,2,5*]

# These authors contributed equally to this work.

*Correspondence author. Email: Dr. Aidong Zhou (aidern0927@smu.edu.cn)

This file includes:

SI Materials and Methods

Materials and Resources Table

Figs. S1-S7

SI References

SI Materials and Methods

GBM tissues

Anonymous archived human GBM specimens were obtained from the Department of Neurosurgery of Nanfang Hospital of Southern Medical University under a protocol approved by the institutional review board. All tissue samples were collected in compliance with the institution's informed consent policy.

Cell culture and treatment

GBM patient-derived GSCs (GSC1023, GSC0603, GSC0910, GSC0917, GSC0111, GSC1209, GSC0709, GSC0718, and GSC1218) were primary cultured in the lab and maintained in DMEM/F-12 (50:50) medium supplemented with B27, EGF (10 ng/mL), and basic fibroblast growth factor (FGF, 10 ng/ml), as we described previously.[1] Only early-passage GSCs were used. U251, U87, 293T, and 293FT cells were cultured in DMEM medium supplemented with 10 % bovine calf serum (Gibco). No cell lines used in this study were found in the database of commonly misidentified cell lines that is maintained by ICLAC and NCBI Biosample. Cell lines were authenticated by short tandem repeat profiling and were routinely tested for mycoplasma contamination. The standard concentrations of the following chemicals for cell treatment are: cycloheximide (CHX), 50 μg/mL; MG132, 20 μM; U0126, 10 μM; TMZ, 100 μM. The information of GBM patients used for GSC establishment was shown in Table S5.

Plasmid construction and mutagenesis

The Myc-tagged full-length FBXO7 and different deletion mutant plasmids were kindly provided by Prof. Judith Stegmüller (Max Planck Institute of Experimental Medicine, Germany). Full length CDS of human *Rbfox2*, *PRMT5,* and *FoxM1b/c* isoforms were amplified by RT-PCR from the cDNA library of GSC1023 cells. *Rbfox2* and *FoxM1b/c* were cloned into p3×Flag-CMV vector, and *PRMT5* was cloned into pcDNA3-HA vectors, respectively. Site-directed mutagenesis of Rbfox2 was introduced using the QuikChange site-directed mutagenesis kit (Agilent Technologies). For lentiviral expression, the CDSs of *Rbfox2* and *FoxM1b/c* were further sub-cloned into pLVX-Neo vector. All plasmids were verified by DNA sequencing. Primers for CDS amplification and mutagenesis are shown in Table S6.

shRNA expression plasmids were constructed by annealing the sense and antisense oligonucleotides of the target sequence and then were cloned into pLKO.1 vector. Oligonucleotides for the construction of shRNA expression plasmids are shown in Table S6.

Lentiviral stable cells

Lentiviral expression plasmids and the packaging mix were transfected into 293FT cells using the lipofectamine 2000 transfection reagent (Invitrogen), according to the manufacturer's instructions. Culture media was changed 8 hours after transfection, and lentiviral particles in the culture medium was collected 36 hours later. GSCs in six-well

plates were infected by lentivirus and then selected by puromycin (2 μg/ml) or neomycin (200 μg/ml) for 1 week.

Immunoprecipitation and mass spectrometry

To identify the proteins associated with FBXO7, the Myc-FBXO7 plasmid was transfected into U251 cells, and cell lysates were immunoprecipitated with an antibody against Myc-Tag. The isolated proteins were separated by SDS-PAGE, followed by sliver staining (ThermoFisher Scientific), according to the manufacturer's instructions. Protein bands of interest were excised and subjected to mass spectrometry analysis (Wininnovate Bio., Shenzhen). MS/MS spectra were searched against the UniProt Homo Sapiens Reference Proteome dataset using Mascot v2.5.1 (Matrix Science).

RNA extraction, transcriptome sequencing and qRT-PCR

We analyzed the changes of gene expression and splicing events after depletion of FBXO7 or Rbfox2 in GSC1023 cells. Total RNA was extracted from the cells using Trizol reagent (Invitrogen) and quantified at an optical density of 260 nm (Nanodrop Technology) with quality assessment using RNA 6000 LabChip kit (Agilent Technologies). Total RNA library was prepared using the Illumina TruSeq RNA Sample Preparation kit (Illumina), according to manufacturer's instruction. The paired-end reads were generated using the Illumina NovaSeq6000 platform (Berry Genomics, Beijing) and then mapped to human genome (hg38).

For qRT-PCR analysis, total RNA was reverse transcribed using PrimeScript™ RT reagent Kit (Takara), according to the manufacturer's instructions. The reverse-transcribed cDNA products were used for qPCR analysis using SYBR® Premix Ex Taq™ (Takara). Primers for qPCR analysis were synthesized by Sangon Biotech (Shanghai). *GAPDH* was used as an internal control. The sequences of primers are shown in Table S6.

Ubiquitination assays

Cellular ubiquitination of Rbfox2 was assessed as we described previously.[1] Briefly, cells were co-transfected with the indicated plasmids for 48 h and then treated with the proteasome inhibitor MG132 (20 μM) for 6 h. Cells were lysed using RIPA lysis buffer (50 mM Tris-base pH 6.8, 150 mM NaCl, 1% NP-40, 0.5% deoxycholic acid, 0.1 % SDS, 10 mM NaF, 10 mM DTT, 0.2 mM Na_3VO_4, 1% cocktail protease inhibitors, 1 mM PMSF). Cell lysates were immunoprecipitated using the indicated antibodies and washed for 3 times by RIPA buffer. To exclude non-specific ubiquitin-modified species from the Rbfox2 complex, we washed the immunoprecipitates for 3 times using a ubiquitination wash buffer (50 mM Tris base pH 6.8, 150 mM NaCl, 1% NP-40, 0.5 % deoxycholic acid, 1 M urea, 1 mM N-ethylmaleimide, and protease inhibitors). For *in vitro* ubiquitination assays, purified Flag-Rbfox2 or Flag-Rbfox2-K249A (5 μM) was incubated with 100 nM UBE1 (E-305, R&D Systems),1 μM UBE2C (NBP2-35090, Novus), 2 μM FBXO7 (P03425, Solarbio), Skp1/Cul1 (E3-410, R&D Systems), 100 μM His-Ubi or His-Ubi-K63 (K63-wildtype only) (R&D Systems), 10 mM Mg^{2+}-ATP

in 50 μL reaction buffer (50 mM Tris PH 7.5, 5 mM $MgCl_2$ and 2 mM DTT) at 37 °C for 1 h. The ubiquitinated proteins were purified by Ni-NTA beads (MedChemExpress), and the eluted proteins were analyzed by immunoblotting.

In vitro methylation assay

The GST-Rbfox2 and GST-Rbfox2-R341A/R441A proteins were expressed in *E. Coli* strain BL21 and purified using GST spin purification kit (ThermoFisher). GST-Rbfox2 or Rbfox2-R341A/R441A protein was incubated with 2 mg of PRMT5 protein, 0.15 mM of S-adenosyl-Methionine at 30 °C for 4 h. Reaction was terminated by addition of the SDS sample buffer, and the result sample was subjected to immunoblotting analysis using an anti-SYM10 antibody.

Extreme Limiting dilution and neurosphere formation assays

We performed *in vitro* limiting dilution assay (LDA) as we described previously.[1] Briefly, GSCs were dissociated to single cells and then plated in 96-well plates at a cell number of 5, 10, 25 or 50 cells per well. Wells with no neurosphere were counted for each group after 10 days. Data was analyzed using software available at http://bioinf.wehi.edu.au/software/elda51. For primary neurosphere formation analysis, dissociated single GSCs were plated at a density of 1 cell/μl, and the spheres that formed after 10 days were counted. For secondary neurosphere assays, established neurospheres were dissociated into single cells and plated at a clonal density of 1 cell/μl,

and the spheres that formed after 10 days were counted. The efficiency of neurosphere formation was calculated by the ratio of spheres/cells.

Immunofluorescence and immunohistochemical analysis

For immunofluorescence (IF) analysis, GBM cells in six-well plates were treated with 4% formaldehyde for 5 min and then treated with 0.5% Triton X-100 for 5 min. The slides were incubated with the indicated primary antibodies, and then incubated with a fluorescent-conjugated second antibody (Life Technologies, 1:1000). Nuclei were co-stained with DAPI. Images were taken using a deconvolution microscope (Zeiss). For immunohistochemical (IHC) staining, tissue slides were deparaffinized, rehydrated through an alcohol series, and then stained with primary antibodies against FBXO7, Rbfox2, CD44, CD9, or ID1 (Table S7 for detailed information for each antibody). We quantified the score of FBXO7 and Rbfox2 staining according to the percentage of cells with positive staining and the staining intensity, as we performed previously.[1] Briefly, we assigned the percentage score as follows: 0 if no cell had staining, 1 if 0-25% of cells had staining, 2 if 25-50%, 3 if 50-75%, 4 if more than 75% of cells had staining. We scored the staining intensity as 0 for negative, 1 for weak, 2 for moderate, and 3 for strong. The total score was obtained by multiplying the percentage score by the intensity score. Three individuals who were blinded to the slides examined and scored each sample.

Intracranial tumor cell injection

All mouse experiments were approved by Institutional Animal Care and Use Committee of Southern Medical University. The sample sizes were justified by statistical considerations and statistical power analyses. The mice were randomly assigned to different experimental groups, and the investigators were blinded to allocation during experiments and outcome assessment. GSCs (5×10^5 cells/mouse) expressing the indicated shRNAs or proteins were injected intracranially into 6- to 8-week-old nude (nu/nu) mice. At the end of the experiment, the mice were humanely killed, and mouse's brain was harvested, fixed in 4% formaldehyde, and embedded in paraffin. Tumor formation was evaluated by hematoxylin and eosin (H&E) staining of mouse tissue sections. Tumor volumes were calculated using the formula $V= (\pi/6) \times a^2 \times b$, where a and b are the tumor's short axis and long axis, respectively.[1] To investigate the effect of FBXO7 depletion on TMZ sensitivity, 1 day after intracranial implantation of GSCs into nude mice (5 mice/group), TMZ (20 mg/kg/day) in a vehicle of dimethyl sulfoxide/polyethylene glycol 300 (Sigma) was injected intraperitoneally every other day for 4 weeks. To analyze mouse survival, animals (5 mice/group) were humanely killed when they were moribund, and the remaining animals were humanely killed 80 d after tumor-cell injection.

Statistics and reproducibility

GraphPad Prism Pro 8.0 software was used for all data analysis. Data are presented as the mean ± standard deviation (S.D.) or standard errors of the mean (S.E.M.). All western blot analysis were repeated three times unless otherwise indicated. For all

representative images, results were reproduced at least three times in independent experiments. For all quantitative data, the statistical test used is indicated in the Figure legends. We assessed differences in the human GBM multiforme data using the Pearson correlation test, the in vitro data between two groups (=2 groups) using the two-tailed Student's t-test, the in vitro data among multiple groups (>2 groups), and the in vivo data using two-way analysis of variance (ANOVA). Mouse survival was analyzed using a Kaplan–Meier model. We considered $P<0.05$ to be significant.

Materials and Resources Table

Table S1. Gene expression changes after FBXO7 depletion.

This table is available online as a separate download.

Table S2. Identification of FBXO7 interacting proteins in GBM cells by MS/MS.

This table is available online as a separate download.

Table S3. Identification of alternative splicing events after Rbfox2 depletion.

This table is available online as a separate download.

Table S4. List of the potential Rbfox2 binding motif (UGCAUG) located downstream or upstream (±300 bp) of the splicing exons.

This table is available online as a separate download.

Table S5. Information of GBM patients used for GSC establishment.

Cell lines	Patient ID	Sex	Age	Pathological type	Molecular features
GSC1023	002109262	Male	58	Glioblastoma (Grade Ⅳ)	P53 (+), EGFR (+++), VEGF (++), IDH1 (-), Ki-67 (+++)
GSC0603	001413801	Male	28	Glioblastoma (Grade Ⅳ)	P53 (+), EGFR (++), VEGF (++), IDH1(-), Ki-67 (++)
GSC0910	001609227	Female	34	Glioblastoma (Grade Ⅳ)	P53 (+), EGFR (++), VEGF (++), IDH1(-), Ki-67 (+++)
GSC0709	001852123	Male	48	Glioblastoma (Grade Ⅳ)	P53(+), EGFR (+++), VEGF (++), IDH1 (-) , Ki-67 (+++)
GSC0917	001714219	Male	51	Glioblastoma (Grade Ⅳ)	P53(-), EGFR (+), VEGF (++), IDH1(-), Ki-67 (++)
GSC0111	001021561	Male	32	Glioblastoma (Grade Ⅳ)	P53 (+), EGFR (++), VEGF (+), IDH1 (-)， Ki-67 (++)
GSC1209	002426252	Female	28	Glioblastoma (Grade Ⅳ)	P53 (-), EGFR (+), VEGF (++), IDH1 (-), Ki-67 (+)
GSC0718	001891673	Male	43	Glioblastoma (Grade Ⅳ)	P53 (-), EGFR (+), VEGF (++), IDH1 (-), Ki-67 (++)
GSC1218	002681257	Male	53	Glioblastoma (Grade Ⅳ)	P53 (-), EGFR (+), VEGF (+), IDH1 (-), Ki-67 (+)

Table S6. Oligonucleotides used in the study.

Name	Sequence (5'-3')
Rbfox2 full-length CDS and mutants	CDS-F: CCCAAGCTTATGGCGGAGGGCGCCCAGCC
	CDS-R: CGGGATCCTCAGTAGGGGGCAAATCGGC
	164R: CGGGATCCTCACTGTGACTGCTGGCCGTCTG
	268R: CGGGATCCTCAACCATTTGCATATGGTGTGA
	164F: CCCAAGCTTCAGTCACAGACACAAAGTAG
	268F: CCCAAGCTTACACCATATGCAAATGGTTGG
	K220A-F: CTCTGCGGGATTCGGGTTCGT
	K220A-R: AATCCCGCAGAGCCACGTTCA
	K239A-F: GGAGGCATTACACGGCACCGT
	K239A-R: TGTAATGCCTCCCTGGCCCTGT
	K249A-F: CCGTGCAATCGAGGTGAATA
	K249A-R: TCGATTGCACGGCCCTCTAC
	R341A-F: TTTCGCAGGAGCCCATTTGAG
	R341A-R: CTCCTGCGAAAGCGGCTGCCGT
	R346A-F: TATACGCAGGTGGCTACAGCCGA
	R346A-R: ACCTGCGTATAAACTCGCCACAG
	R441A-F: ACAGCGCATTTGCCCCCTACTG
	R441A-R: CAAATGCGCTGTAGCCACCTCG
PRMT5 full-length CDS cloning	PRMT5-F: CGGGATCCAGAAAGATGGCGGCGATGGC
	PRMT5-R: CCGCTCGAGACACTTGGCACGCAGGGCTA
FoxM1b/FoxM1c isoform CDS cloning	FoxM1-F: ggaattcAATGGAGAGTGAAAACGCAGATTC
	FoxM1-R: cgggatccATGGTGGACAGCTTGAGCACAG
FBXO7-shRNAs	Sh1-F: CCGGGCCACATTCATTAGAGACCTTCTCGAGAAGGTCTCTAATGAATGTGGCTTTTTG
	Sh1-R: AATTCAAAAAGCCACATTCATTAGAGACCTTCTCGAGAAGGTCTCTAATGAATGTGGC

	Sh2-F: CCGGGCTGACTGTTCTGATGCCAATCTCGAGATTGGCATCAGAACAGTCAGCTTTTTG
	Sh2-R: AATTCAAAAAGCTGACTGTTCTGATGCCAATCTCGAGATTGGCATCAGAACAGTCAGC
Rbfox2-shRNAs	Sh1-F: CCGGCGGGTTCGTAACTTTCGAGAACTCGAGTTCTCGAAAGTTACGAACCCGTTTTTG
	Sh1-R: AATTCAAAAACGGGTTCGTAACTTTCGAGAACTCGAGTTCTCGAAAGTTACGAACCCG
	Sh2-F: CCGGGTATATGGTCCGGAGTTATATCTCGAGATATAACTCCGGACCATATACTTTTTG
	Sh2-R: AATTCAAAAAGTATATGGTCCGGAGTTATATCTCGAGATATAACTCCGGACCATATAC
PRMT5-shRNAs	Sh1-F: CCGGGCCCAGTTTGAGATGCCTTATCTCGAGATAAGGCATCTCAAACTGGGCTTTTTG
	Sh1-R: AATTCAAAAAGCCCAGTTTGAGATGCCTTATCTCGAGATAAGGCATCTCAAACTGGGC
	Sh2-F: CCGGCCCATCCTCTTCCCTATTAAGCTCGAGCTTAATAGGGAAGAGGATGGGTTTTTG
	Sh2-R: AATTCAAAAACCCATCCTCTTCCCTATTAAGCTCGAGCTTAATAGGGAAGAGGATGGG
FoxM1-shRNAs	Sh1-F: CCGGGCCAATCGTTCTCTGACAGAACTCGAGTTCTGTCAGAGAACGATTGGCTTTTTG
	Sh1-R: AATTCAAAAAGCCAATCGTTCTCTGACAGAACTCGAGTTCTGTCAGAGAACGATTGGC
	Sh2-F: CCGGGCCCAACAGGAGTCTAATCAACTCGAGTTGATTAGACTCCTGTTGGGCTTTTTG
	Sh2-R: AATTCAAAAAGCCCAACAGGAGTCTAATCAACTCGAGTTGATTAGACTCCTGTTGGGC
sqRT-PCR	FoxM1-F: AATGGCAAGGTCTCCTTCTGG
	FoxM1-R: TTCGGTTTTGATGGTCATGTTC
	MTA1-F: AAAAGCGTGTCCAGCGTGCTC
	MTA1-R: TGTCCGTGGTTTGCCAGACC
	POSTN-F: TTGTTCGTGGTAGCACCTTC
	POSTN-R: GTAATGATTCGTTCTTCTCGTG
	CKIδ-F: AAAGTGAGTATGCGGCTGCA
	CKIδ-R: AACAGAGTAGATCAGCCATGC
	SCARB1-F: ACATTCTACACTCAGCTGGTG
	SCARB1-R: TGTGCAACAGGCACATGGCA
	FYN-F: ACACTTCAGCAGCTTGTACAAC

	FYN-R: TTCTTCTCCAGACACAACGAAC
	CARM1-F: TTGAGCAGTGTTATTGCCAGT
	CARM1-R: AACTGGCTGTTGACTGCATAG
	NCOR2-F: TTCAATATGCCCGCCATCACC
	NCOR2-R: AGTGCACTGAGGAGACAGAG
qRT-PCR	CD44-F: ATAGCACCTTGCCCACAATGG
	CD44-R: TGTTGGATGTGAGGATGTACAC
	CD9-F: AAGTGCATCAAATACCTGCTG
	CD9-R: TAGACTCCTGTGTAGAAGCTG
	TIMP3-F: TTCTGCAACTCCGACATCGTG
	TIMP3-R: AAGCTTCCGTATGGATGTACTG
	ID1-F: TGAACTCGGAATCCGAAGTTGG
	ID1-R: TTCAGCGACACAAGATGCGATC
	FoxM1-F: TGCAGCTAGGGATGTGAATCTTC
	FoxM1-R: GGAGCCCAGTCCATCAGAACT
	FoxM1b-F: CCAGGTGTTTAAGCAGCAGA
	FoxM1b-R: TCCTCAGCTAGCAGCACCTTG
	FoxM1c-F: CAATTGCCCGAGCACTTGGAATCA
	FoxM1c-R: TCCTCAGCTAGCAGCACCTTG
	Mta1-l-F: AAAAGCGTGTCCAGCGTGCTC
	Mta1-l-R: ATCAAGAGGCGCTTCTTCATG
	Mta1-s-F: AAAAGCGTGTCCAGCGTGCTC
	Mta1-s-R: TGGTTTGCCAGACCCGTCCA
	Postn-l-F: TAACTGAAGTGATCCATGGAG
	Postn-l-R: GTAATGATTCGTTCTTCTCGTG
	Postn-s-F: ACTGAAGGATTAAAAAATACACC
	Postn-s-R: GTAATGATTCGTTCTTCTCGTG

RIP-qPCR	FoxM1-RIP-F: ACAATTGCCCGAGCACTTGGA
	FoxM1-RIP-R: ACATCACATCTTGTCTCTGTCC
	Mta1-RIP-F: AACATGAAGAAGCGCCTCTTG
	Mta1-RIP-R: TTCACCAAGGGACCAGTGATG
	Postn-RIP-F: TAACTGAAGTGATCCATGGAG
	Postn-RIP-R: TCTGACCAGCTAATAAGAACTC

Table S7. Antibodies used in the study.

Antibody	Company	Cat. No.	Dilution
FBXO7	Abcam	ab84129	WB: 1:1000<br>RRID：AB_1924983
FBXO7	Abcam	ab167278	WB: 1:500; IF: 1:100
FBXO7	SAB	30934	IHC: 1:100<br>RRID：AB_911509
Rbfox2	Abcam	ab264154	WB: 1:1000; IHC: 1:100
Rbfox2	ProteinTech	12498-1-AP	WB: 1:1500; IF: 1:100<br>RRID：AB_2877861
CD44	ProteinTech	15675-1-AP	WB: 1:1000; IF: 1:100<br>RRID：AB_2076198
CD44	BioLegend	338805	FACS<br>RRID：AB_1501202
CD9	ProteinTech	60232-1-Ig	WB: 1:500, IF: 1:50<br>RRID：AB_11232215
ID1	Santa Cruz	sc-133104	WB: 1:1000<br>RRID：AB_2122863
Vimentin	Santa Cruz	sc-6260	WB: 1:1000, IF: 1:200<br>RRID：AB_628437
Olig2	ProteinTech	66513-1-Ig	WB: 1:1000, IF: 1:50<br>RRID：AB_2881876
Sox2	ProteinTech	66411-1-Ig	WB: 1:1000<br>RRID：AB_2881783
PRMT5	Santa Cruz	sc-376937	IP: 1:200, WB: 1:1000, IF: 1:200<br>RRID：AB_2904201
FoxM1	Santa Cruz	sc-376471	IF/IHC: 1:300, WB: 1:500<br>RRID：AB_11150135
Ki-67	Santa Cruz	sc-23900	IHC: 1:200<br>RRID：AB_627859
Flag-Tag	Sigma	F1804	IP: 1:500, WB: 1:5000<br>RRID：AB_262044
HA-Tag	CST	3724S	IP: 1:500, WB: 1:3000
Myc-Tag	CST	2276S	IP: 1:500, WB: 1:3000
SYM10	Merck Millipore	07-412	WB: 1:1000<br>RRID：AB_310594
Tubulin	Ray antibody	RM2007	WB: 1:1000<br>RRID：AB_2934267

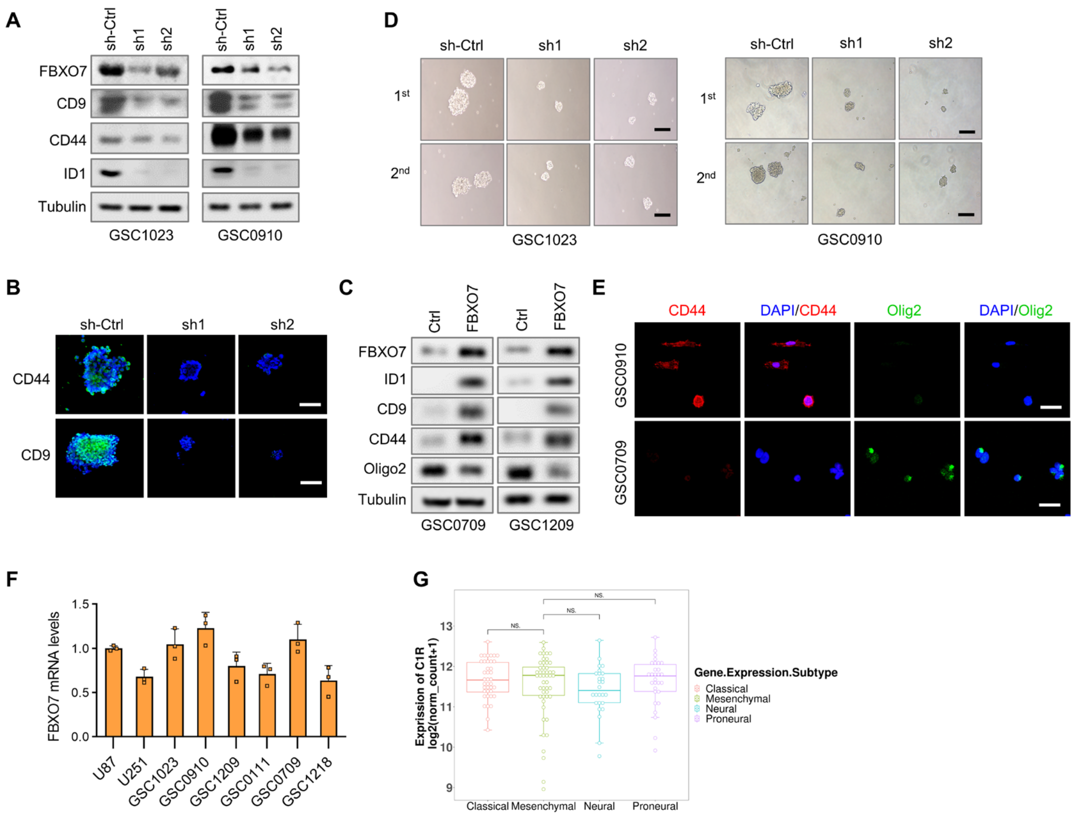

Figure S1. FBXO7 promotes GBM MES properties and tumorigenesis. **A**, Cell lysates of GSC1023 and GSC0910 cells stably expressing *FBXO7* shRNAs were analyzed by immunoblotting using the indicated antibodies. **B**, Immunofluorescence (IF) staining detected the expression of CD44 and CD9 in GSC1023 cells expressing *FBXO7* shRNAs. Representative images were shown. Scale bar, 200 μm. **C,** Cell lysates of GSC0709 and GSC1209 cells stably expressing FBXO7 were analyzed by immunoblotting using the indicated antibodies. **D**, Representative photographs of primary (1st) or secondary (2nd) neurosphere formation of GSC1023 and GSC0910 cells stably expressing *FBXO7* shRNAs. Scale bar, 500 μm. **E**, IF co-staining of CD44 and Olig2 in the MES-like GSC0910 cells and PN-like GSC0709 cells. Representative images were shown. Scale bar, 50 μm. **F**, *FBXO7* mRNA levels were evaluated by qRT-PCR in GBM cell lines and patients-derived GSCs (mean ± S.E.M., n=3 independent experiments). *GAPDH* was used as an internal control. **G**, Relative expression of *FBXO7* mRNA in different GBM subtypes from the TCGA datasets.

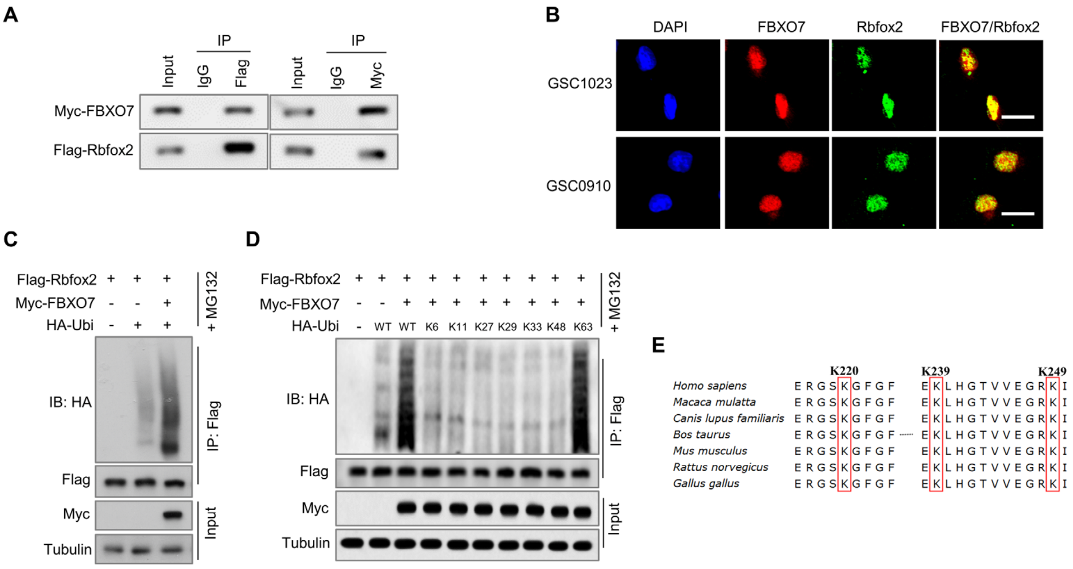

Figure S2. FBXO7 interacts with Rbfox2 and promotes K63-linked ubiquitination of Rbfox2. A, 293T cells were transfected with Myc-FBXO7 and Flag-Rbfox2 plasmids, and cell lysates were incubated with an anti-Myc or anti-Flag antibody. The immunoprecipitated proteins were subjected to immunoblotting using the indicated antibodies. **B**, IF Co-staining of FBXO7 and Rbfox2 in GSC1023 and GSC0910 cells. Scale bar, 50 μm. **C**, 293T cells were transfected with Flag-Rbfox2, Myc-FBXO7 and HA-Ubi, and then treated with MG132 for 6h before harvest. Cell lysates were immunoprecipitated using an anti-Flag antibody and then analyzed by immunoblotting. **D**, 293T cells were transfected with Flag-Rbfox2, Myc-FBXO7, and HA-Ubi or different ubiquitin mutants. Cell lysates were immunoprecipitated using an anti-Flag antibody and then analyzed by immunoblotting. **E**, Amino acid sequence conservation of Rbfox2 in different species.

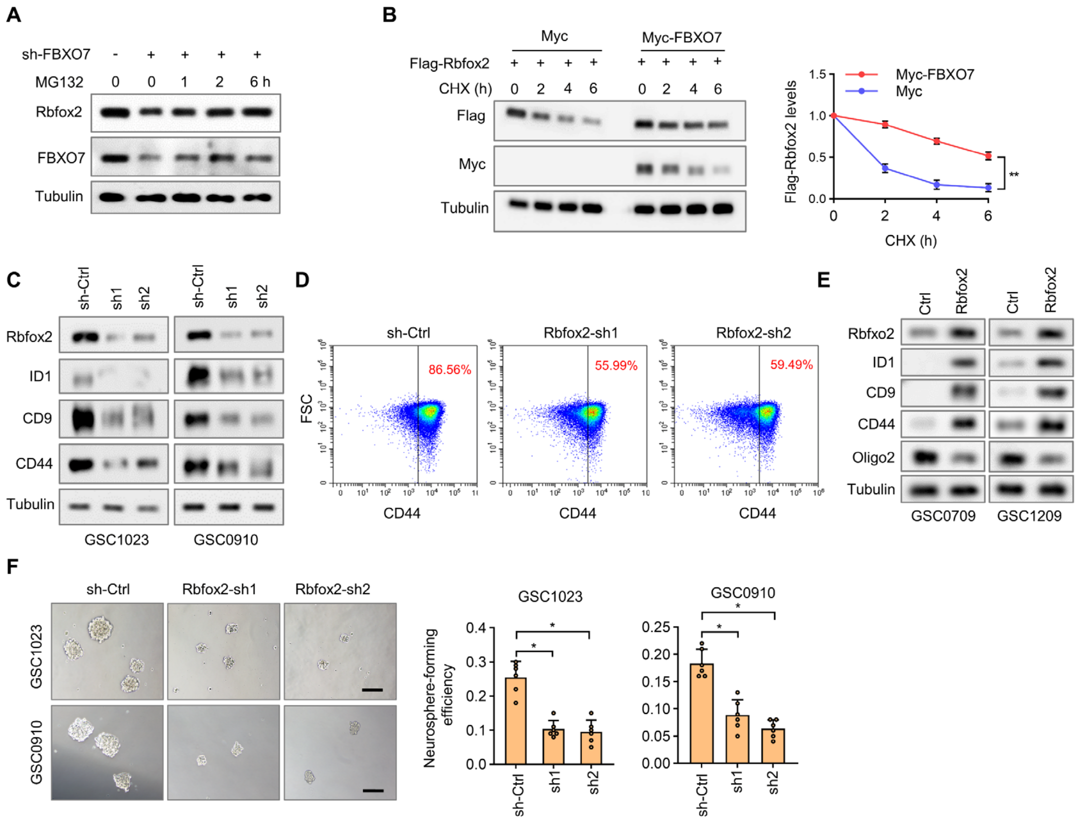

Figure S3. Rbfox2 is stabilized by FBXO7 and mediates FBXO7-induced GBM MES transformation and tumorigenesis. **A**, GSC1023 cells expressing *FBXO7* shRNA were treated with MG132 for the indicated intervals, and cell lysates were analyzed by immunoblotting. **B**, 293T cells were transfected with Flag-Rbfox2 and Myc-FBXO7 plasmids and then treated with CHX for the indicated time intervals. Cell lysates were analyzed by immunoblotting. The band intensity was quantified and the results are expressed as Flag-Rbfox2 levels relative to control (mean ± S.D., n=3 independent experiments, paired Student's t-test). **C**, Cell lysates of GSC1023 and GSC0910 cells expressing *Rbfox2* shRNAs were analyzed by immunoblotting using the indicated antibodies. **D**, FACS analysis of CD44 in GSC1023 expressing *Rbfox2* shRNAs. **E**, Cell lysates of GSC0709 and GSC1209 cells stably expressing Rbfox2 were analyzed by immunoblotting using the indicated antibodies. **F**, Neurosphere formation analysis of GSC1023 and GSC0910 cells expressing *Rbfox2* shRNAs. Representative images were shown (left). Scale bar, 500μm. The neurosphere formation

efficiency (spheres/cells plated) was calculated (Right panel, mean ± S.E.M., n=6 independent experiments, two-tailed Student's t-test). $^{*}P<0.05$.

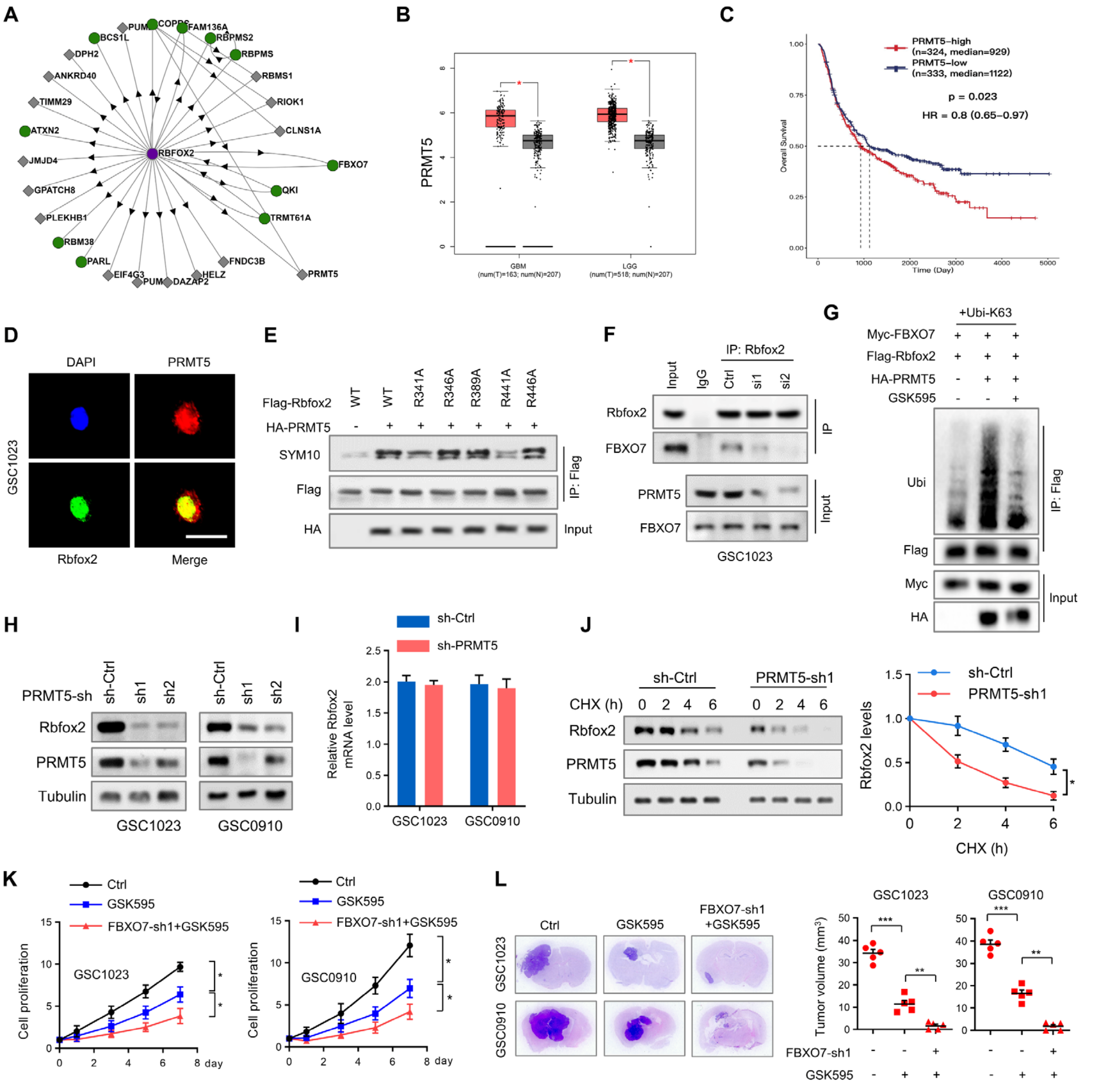

Figure S4. PRMT5-mediated arginine dimethylation of Rbfox2 promotes Rbfox2 ubiquitination by FBXO7. **A**, Scheme shows the potential interacting partners of Rbfox2 using the Bioplex dataset (https://bioplex.hms.harvard.edu/). **B**, TCGA datasets demonstrates the expression of PRMT5 mRNA in normal brain tissue, low grade glioma (LGG) and GBM. **C**, TCGA datasets demonstrates that high-level PRMT5 predicts poor overall survival in glioma. **D**, IF co-staining of PRMT5 and Rbfox2 in GSC1023 cells. Scale bar, 20 μm. **E**, 293T cells were transfected with HA-PRMT5 and different Rbfox2 mutant constructs. The cell lysates were immunoprecipitated with an anti-Flag antibody and then were subjected to immunoblotting using an anti-SYM10 antibody. **F**, GSC1023 cells were transfected with PRMT5 siRNAs, and cell lysates

were immunoprecipitated using an anti-Rbfox2 antibody. The resultant precipitates were analyzed by immunoblotting using the indicated antibodies. **G,** 293T cells were transfected with HA-PRMT5, Myc-FBXO7, Flag-Rbfxo2, and Ubi-K63, and then were treated with GSK3326595 for 24h. Cell lysates were immunoprecipitated with an anti-Flag antibody and then analyzed by immunoblotting. **H** and **I,** Cell lysates of GSC1023 and GSC0910 cells expressing PRMT5 shRNAs were analyzed by immunoblotting (H) and quantitative RT-PCR (I). For qRT-PCR, values were normalized to control shRNA (mean ± S.E.M., n=2 independent experiments). **J**, GSC1023 cells expressing PRMT5 shRNA were treated with CHX for the indicated time intervals, and cell lysates were analyzed by immunoblotting using PRMT5 and Rbfox2 antibodies. Band intensities of Rbfox2 were quantified and the results are expressed as Rbfox2 levels relative to control (mean ± S.D., n=3 independent experiments, paired Student's t-test, right panel). $^{*}P<0.05$. **K**, GSC1023 and GSC0910 cells with FBXO7 depletion or not were treated with GSK3326595 (GSK595), and cell growth were determined by CCK8 assays. values were normalized to control (mean ± S.E.M., n=3 independent experiments). *P<0.05. **L**, GSC1023 and GSC0910 cells (5×10^5 cells/mouse) with FBXO7 depletion or not were intracranially injected into nude mice. The mice were treated with GSK3326595 (50 mg/kg, oral gavage, daily) 7 days after cell implantation, and tumor growth was assessed. The H&E-stained sections show representative tumor xenografts. Tumor volumes were calculated (mean ± S.D., n=5 mice for each group, One-way ANOVA test). $^{**}P<0.05$, $^{***}P<0.05$.

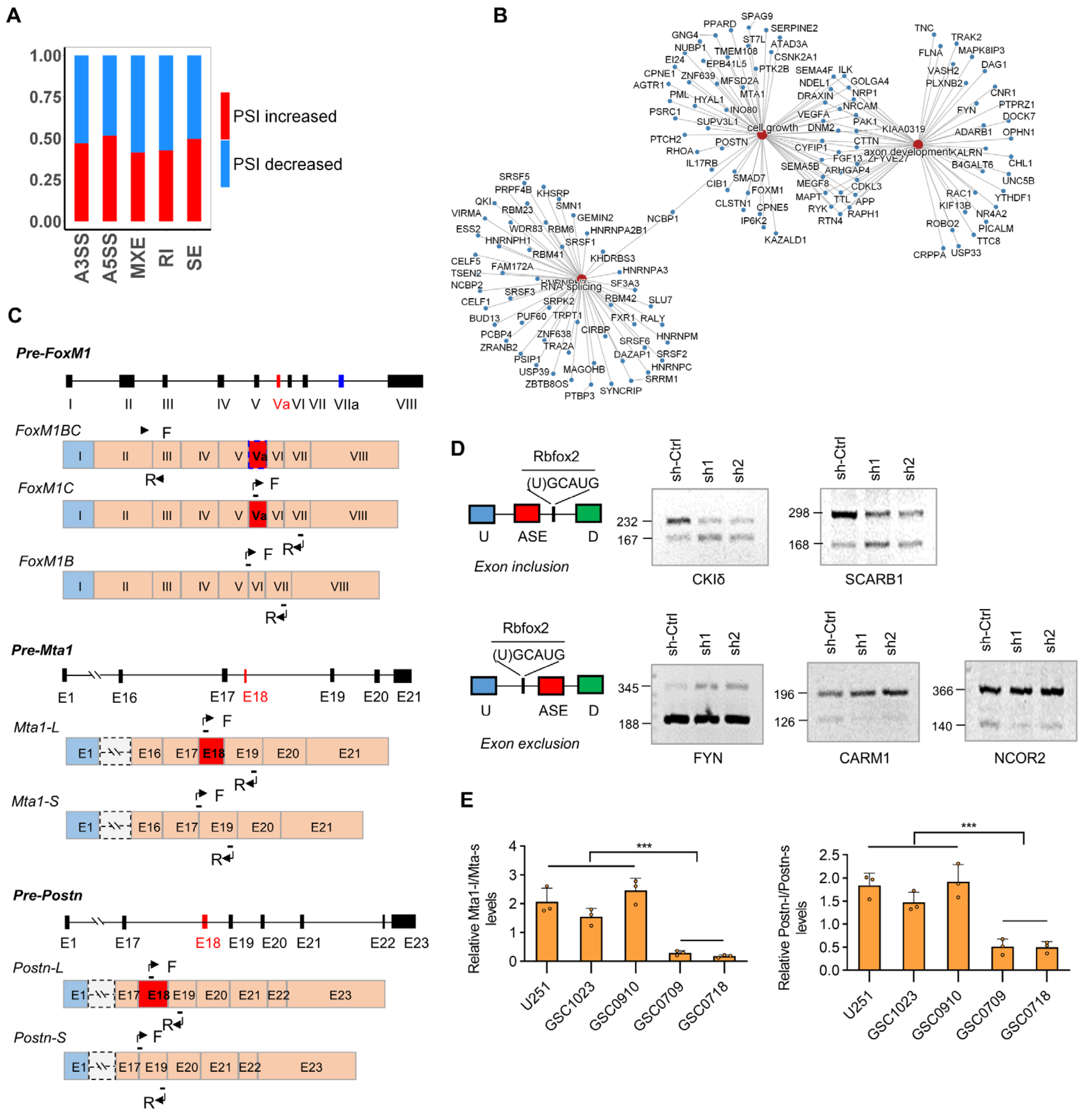

Figure S5. FBXO7 controls Rbfox2-mediated alternative splicing of MES genes. A, The relative fraction of each AS event affected positively or negatively by Rbfox2. **B**, Functional association network of Rbfox2-regulated AS target genes were analyzed by the STRING database. **C**, Exon organization of different isoforms of *FoxM1*, *Mta1*, and *Postn* gene. The alternative exon of each gene was indicated in red box. Primers used for isoform-specific qRT-PCR amplification were indicated by arrows. **D**, Semi-quantative RT-PCR detected the expression of the spliced isoforms of example genes after Rbfox2 depletion. These genes include *CKIδ* and *SCARB1*, in which the Rbfox2 motif locates downstream of the spliced exons, and *FYN*, *CARM1* and *NCOR2*, in which the Rbfox2 motif locates upstream of the spliced exons. **E**, The levels of different *Mta1*

and *Postn* splicing isoforms in a panel of GSCs were determined by qRT-PCR. Values were expressed as Mta1-l/Mta1-s (left) and Postn-l/Postn-s (right) levels, respectively. *GAPDH* was used as an internal control (mean ± S.E.M., n=3 independent experiments). ***P<0.01.

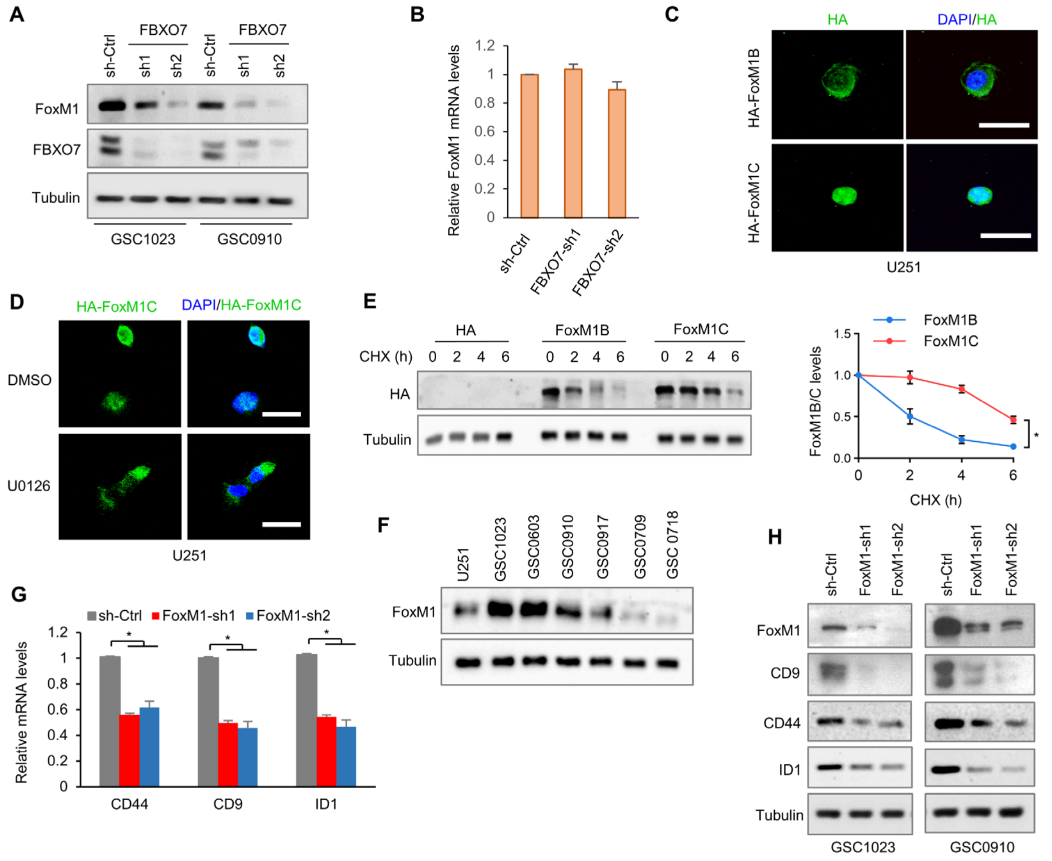

Figure S6. The FBXO7-Rbfox2 axis-mediated splicing of FoxM1 promotes GBM MES transformation and tumorigenesis. **A**, Cell lysates of GSC1023 and GSC0910 cells expressing *FBXO7* shRNAs were analyzed by immunoblotting. **B**, Total *FoxM1* mRNA levels in GSC1023 cells expressing *FBXO7* shRNAs were detected by qRT-PCR (mean ± S.E.M., n=3 independent experiments). **C**, U251 cells were transfected with HA-FoxM1b or HA-FoxM1c, and IF staining was performed using an anti-HA antibody. Representative photographs were shown. Scale bar, 50 µm. **D**, U251 cells were transfected with HA-FoxM1c and then were treated with a MEK1/2 inhibitor U0126 (10µM) for 2h. Cells were immunostained with an anti-HA antibody. Scale bar, 50 µm. **E**, 293T cells expressing FoxM1B or FoxM1C were treated with CHX for the indicated time intervals, and cell lysates were analyzed by immunoblotting. Band intensity of HA was quantified and the results are expressed as levels of HA-FoxM1C vs HA-FoxM1B (mean ± S.D., n=3 independent experiments, paired Student's t-test).

*P<0.05. **F**, Cell lysates of U251 cells and different GSCs were analyzed by immunoblotting using an anti-FoxM1 antibody. **G**, qRT-PCR analysis of indicated genes in GSC1023 cells expressing *FoxM1* shRNAs (mean ± S.E.M., n=3 independent experiments). *P <0.05. **H**, Cell lysates of GSC1023 and GSC0910 cells expressing *FoxM1* shRNAs were analyzed by immunoblotting using the indicated antibodies.

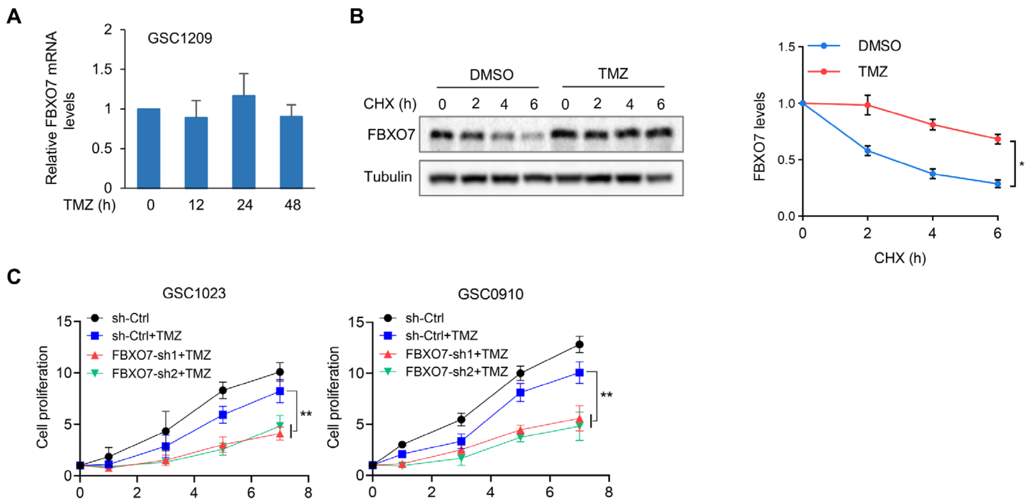

Figure S7. FBXO7 is induced by TMZ and promotes chemoresistance in GBM. **A**, GSC1209 cells were treated with TMZ (100 μM) for the indicated time intervals, and *FBXO7* mRNA levels were detected by qRT-PCR (mean ± S.D., n=3 independent experiments). **B**, GSC1209 cells were treated with DMSO or TMZ (100 μM), and then treated with CHX for the indicated time intervals. Cell lysates were subjected to immunoblotting. FBXO7 Band intensity was quantified and the results are expressed as levels of TMZ vs DMSO (mean ± S.D., n=3 independent experiments, paired Student's t-test). *P <0.05. **C**, GSC1023 and GSC0910 cells expressing sh-Ctrl or *FBXO7* shRNAs were treated with TMZ (100 μM) for the indicated times, and cell viabilities were determined by XTT assays (mean ± S.D., n=3 independent experiments, paired Student's t-test). P values were analyzed by comparing TMZ along versus the combination of TMZ plus FBXO7 depletion. **P <0.01.

References

[1] A. Zhou, K. Lin, S. Zhang, Y. Chen, N. Zhang, J. Xue, Z. Wang, K. D. Aldape, K. Xie, J. R. Woodgett, S. Huang, *Nature cell biology* **2016**, *18* (9), 954, https://doi.org/10.1038/ncb3396.
